# Effects of acute caffeine supplementation on physical, physiological, and sport-specific performance in female volleyball players: a systematic review and three-level meta-analysis

**DOI:** 10.3389/fnut.2026.1880118

**Published:** 2026-07-08

**Authors:** Zike Zhang, Youheng Wang, Zhe Lu, Fanhao Meng, Wenxin Du, Anqi Chen, Bitai Wu, Zhicong Zhang, Yunxiang Sun, Jiali Lai, Qi Liu, Bichen Li, Song Hu, Yimin Wang

**Affiliations:** 1 School of Physical Education, Shaanxi Normal University, Xi'an, China; 2 Department of Sport and Sport Science, Exercise and Human Movement Science, University of Freiburg, Freiburg, Germany; 3 Department of Physical Education, College of Education, Zhejiang University, Hangzhou, Zhejiang, China; 4 Graduate School of Shandong Sport University, Jinan, China; 5 School of Physical Education and Sports Science, Hengyang Normal University, Hengyang, Hunan, China

**Keywords:** ergogenic aids, fatigue, jump performance, neuromuscular function, women athletes

## Abstract

**Introduction:**

Previous research has suggested that caffeine can enhance performance in volleyball. However, most meta-analyses on caffeine supplementation in volleyball have primarily focused on male athletes or mixed-sex samples. To date, no meta-analysis has specifically examined the effects of caffeine in female volleyball players. Therefore, the present study aimed to synthesize the available evidence to provide a cautious and preliminary evaluation of the effects of caffeine on multidimensional performance outcomes in female volleyball athletes.

**Methods:**

A systematic search was conducted in March 2026 across PubMed, Web of Science, the Cochrane Library, and ProQuest. The review was registered in PROSPERO (CRD420261355948). Randomized controlled trials examining the effects of caffeine intake on performance in female volleyball players were included. Methodological quality and risk of bias were assessed using the PEDro scale and the RoB 2 tool, and the certainty of evidence was evaluated using the GRADE approach.

**Results:**

Five studies were included, comprising a total of 66 female athletes. Overall methodological quality was high, although some risk of bias was identified. According to the GRADE assessment, the overall certainty of evidence was very low. The exploratory meta-analysis did not provide clear evidence supporting an ergogenic effect of caffeine supplementation on sport-specific performance (spike performance), physical performance (jump height, change of direction, handgrip strength, and power output), or physiological outcomes (fatigue- and heart rate-related parameters) in female volleyball players (*p* > 0.05). In addition, caffeine intake was associated with potential adverse effects, including hand tremors, anxiety, and excessive stimulation.

**Conclusion:**

Based on the currently limited evidence, acute caffeine ingestion (2–6.4 mg/kg) did not show a consistent ergogenic benefit multiple performance outcomes in female volleyball players and may be associated with potential adverse effects, such as hand tremors and anxiety. Therefore, the findings should be interpreted cautiously as preliminary and exploratory evidence rather than as a basis for definitive practical recommendations.

**Systematic review registration:**

This systematic review was registered in PROSPERO (Registration number: CRD420261355948); URL: https://www.crd.york.ac.uk/PROSPERO/view/CRD420261355948.

## 1 Introduction

Volleyball, as a widely practiced intermittent team sport (1), is characterized by the alternation of high-intensity activity and brief recovery periods (2). A single match typically lasts 60–90 min, during which players are required to perform frequent high-intensity actions such as rapid changes of direction, jumping, and spiking, while maintaining sustained attentional focus (3, 4). Among various physical attributes, jumping ability and upper-body strength are considered important performance determinants in volleyball (5, 6). These capacities are closely associated with the execution quality of key technical skills, such as spiking and blocking, and to a certain extent, influence overall match performance and outcomes (3, 7). Previous studies have reported that female volleyball players perform an average of approximately 45 jumps per match, with individual counts reaching up to 73 jumps (8). Under simulated match conditions, players frequently experience substantial trunk accelerations, with 361–297 and 97–38 high-impact events exceeding 4G and 6G thresholds, respectively, indicating a considerable volume of high-intensity accelerative and impact loading during play (9). Such repetitive high-load jump–landing cycles and rapid accelerations are likely to induce acute exercise-induced fatigue, which may impair performance and increase the risk of musculoskeletal injuries, including anterior cruciate ligament (ACL) rupture, patellar tendinopathy, and ankle joint injuries (10–15). Consequently, strategies to mitigate fatigue and maintain performance have become increasingly important in volleyball. Given the substantial physiological demands and intermittent high-intensity nature of volleyball, athletes often adopt targeted nutritional strategies to support training adaptations, maintain performance, and enhance recovery as well as repeated exercise capacity (16, 17). Among various ergogenic aids, caffeine is one of the most commonly used due to its well-established efficacy in enhancing endurance and exercise performance across multiple sports (18, 19).

Since its removal from the World Anti-Doping Agency (WADA) list of prohibited substances, while remaining under the WADA Monitoring Program, caffeine has been widely investigated and is considered to have ergogenic potential, contributing to its widespread use across various sports (17, 20–23). As a non-selective adenosine receptor antagonist, caffeine can attenuate the inhibitory effects of adenosine on the central nervous system, thereby enhancing central excitability and increasing arousal during exercise. In addition, caffeine may promote calcium release from the sarcoplasmic reticulum (24, 25), which can enhance force production, motor unit recruitment, and rate coding of large muscle groups (26, 27). It has also been suggested to reduce perceived exertion, alleviate muscle soreness, and lower the risk of sports-related injuries (28–30) Collectively, these multifaceted effects may provide a physiological basis for the potential enhancement of performance in female volleyball athletes.

A preliminary statistical analysis of the existing literature indicated that the sex distribution of samples in studies examining caffeine and volleyball athletes is relatively balanced, with female and male samples (including independent samples comprising both sexes) each accounting for approximately 50% (31–40). However, existing meta-analytic evidence on caffeine intake and volleyball performance is primarily based on studies involving male or mixed-sex samples (41, 42), whereas systematic evidence specifically focusing on female volleyball athletes remains markedly limited. This indicates a clear sex imbalance in the current synthesis of evidence regarding caffeine use in volleyball. Given that fluctuations in estrogen levels across the menstrual cycle may influence both performance in female volleyball athletes and the ergogenic effects of caffeine (18, 43, 44), and that oral contraceptive use may alter caffeine metabolism, prolong its half-life and duration of action in the body (45), directly extrapolating findings from studies including male samples to female populations presents clear limitations and increases uncertainty regarding the application of caffeine in female volleyball athletes. Accordingly, several researchers have emphasized the necessity of sex-specific investigations and highlighted the persistent sex differences in the literature (18–20, 46–48).

Given these limitations, the effects of caffeine on female volleyball athletes remain to be further elucidated. To comprehensively assess these effects, the present study adopted the classification approach of Zhang et al. (18), categorizing outcome measures into sport-specific technical performance, physical performance, and physiological responses. This framework was used to summarize the potential external and internal responses to acute caffeine ingestion in female volleyball athletes. Accordingly, we conducted a systematic review and exploratory meta-analysis focusing on female volleyball athletes to summarize the evidence on the acute effects of caffeine on sport-specific technical performance, physical performance, and physiological responses, and to preliminarily examine the potential moderating roles of menstrual cycle phase, caffeine dosage, and timing of ingestion. Specifically, this study sought to answer the following research question: Compared with placebo or control conditions, what are the acute effects of caffeine intake on sport-specific technical performance, physical performance, and physiological responses in female volleyball athletes?

## 2 Materials and methods

### 2.1 Search strategy

This systematic review and meta-analysis was conducted in accordance with the Preferred Reporting Items for Systematic Reviews and Meta-Analyses (PRISMA) 2020 guidelines (Supplementary Table 1) (49) and was prospectively registered in PROSPERO (CRD420261355948). A systematic literature search was conducted in PubMed, Web of Science, the Cochrane Library, and ProQuest from database inception to March 2026. The search strategy incorporated both Medical Subject Headings (MeSH) and free-text terms related to key concepts of caffeine and volleyball, developed and adapted using Boolean operators and wildcards: (concept 1) (“Caffeine”[MeSH Terms] OR caffeine [Title/Abstract] OR coffee [Title/Abstract] OR ergogenic aid*[Title/Abstract] OR stimulant*[Title/Abstract] OR supplement [Title/Abstract]) AND (concept 2) (“volleyball”[MeSH Terms] OR volleyball [All Fields] OR “volleyball”[Title/Abstract] OR volleyball player*[Title/Abstract] OR volleyball athlete*[Title/Abstract] OR team sport*[Title/Abstract]). All retrieved records were exported to a CSV file for deduplication in EndNote X9 (Clarivate Analytics, New York, NY, United States). In addition, the reference lists of all included studies were manually screened to identify any potentially eligible studies. Study identification, screening, and final selection were independently completed by two reviewers (ZZK and WYH), with disagreements resolved through discussion.

### 2.2 Inclusion and exclusion criteria

Based on the PICOS framework, the inclusion and exclusion criteria were defined as follows: (a) Population: Studies involving female volleyball athletes were included. For studies comprising male participants, mixed-sex samples, or unspecified sport disciplines, only data specific to female volleyball athletes were extracted. Studies that did not report female-specific data were excluded from the review (31, 50). Studies involving wheelchair athletes or athletes with disabilities were also excluded; (b) Intervention: Nutritional interventions in which caffeine was the primary active ingredient were included, with no restrictions on dosage, timing, or mode of administration. Studies evaluating chronic caffeine intake in longitudinal designs, multi-ingredient supplements in which caffeine was not the principal active component, or interventions lacking a clearly defined caffeine supplementation protocol were excluded; (c) Comparator: Eligible studies were required to include a placebo-controlled group under comparable experimental conditions. Studies without placebo control were excluded; (d) Outcomes: Studies reporting at least one quantified outcome related to sports performance (e.g., vertical jump performance or physiological parameters) were included. Studies that did not report any performance-related outcomes specific to female volleyball athletes were excluded. (e) Study design: Only blinded controlled trials, including randomized crossover trials and randomized parallel-group trials, were considered eligible for inclusion. Unpublished data, grey literature, systematic reviews, and meta-analyses were excluded. The eligibility of each primary study was independently assessed according to the predefined inclusion and exclusion criteria, regardless of whether it had previously been included in published systematic reviews or meta-analyses.

### 2.3 Data extraction and transformation

Data extraction was independently conducted by two reviewers involved in the screening phase using a standardized data extraction form developed in Microsoft Excel prior to full-text screening. The following information was extracted: study characteristics (author and year of publication), participant characteristics, details of the exercise intervention, and clinical outcome measures. If data were missing or presented only in graphical form, the corresponding authors were contacted to obtain the required information. If no response was received and data were available only graphically, relevant data were extracted using WebPlotDigitizer 4.1 software.^1^ Studies with missing data that could not be obtained were excluded from the analysis.

In this review, for studies that directly reported post-intervention means and standard deviations of the relevant outcomes (caffeine vs. placebo), these data were extracted as presented. For studies in which outcomes were reported only as pre- and post-intervention values, baseline and post-intervention data were extracted, and the mean change and corresponding standard deviation were derived using established formulas: The mean difference ( $M_{\text{diff}}$ ) between the two time points was calculated using the following formula (51):


$$M_{\text{diff}}=M_{\text{post}}-M_{\mathrm{pre}},$$

where $M_{\text{diff}}$ denotes the raw mean difference, $M_{\text{post}}$ the reported post-intervention mean, and $M_{\mathrm{pre}}$ the reported pre-intervention mean.

The standard deviation of the mean difference ( ${\mathrm{SD}}_{\text{diff}}$ ) was then calculated as follows (51):


$${\mathrm{SD}}_{\text{diff}}=\sqrt{{{\mathrm{SD}}_{\mathrm{pre}}}^{2}+{{\mathrm{SD}}_{\text{post}}}^{2}-2r\times {\mathrm{SD}}_{\mathrm{pre}}\times {\mathrm{SD}}_{\text{post}}},$$

Here, r denotes the pre–post correlation coefficient. In accordance with the Cochrane Handbook, a conservative value of 0.5 was assumed (51). The estimated mean change and its corresponding standard deviation were subsequently used to calculate the standardized mean difference (SMD) for the meta-analysis.

### 2.4 Quality assessment of included studies

The risk of bias was independently assessed by two reviewers, with disagreements resolved through discussion when possible. If consensus could not be reached, a third reviewer arbitrated the decision. The Cochrane risk-of-bias tool for randomized trials (RoB 2) (52) was used to assess risk of bias across five domains: bias arising from the randomization process, bias due to deviations from intended interventions, bias due to missing outcome data, bias in measurement of the outcome, and bias in selection of the reported result. Additionally, the Physiotherapy Evidence Database (PEDro) scale was employed to evaluate the methodological quality and risk of bias of the included studies. The PEDro scale rates studies on a 0–10 scale, with scores ≥6 indicating high quality, scores of 4–5 indicating moderate quality, and scores ≤3 indicating low quality (53).

### 2.5 Evidence grading

The certainty of evidence was evaluated using the GRADE framework (54) and categorized as high, moderate, low, or very low. The assessment was conducted according to the following predefined criteria: (a) Risk of bias: Certainty was downgraded by one level for outcomes judged as having “some concerns” and by two levels for those at “high risk” of bias; (b) Inconsistency: Certainty was downgraded by one level when heterogeneity was moderate (*I*^2^ = 25–75%) and by two levels when heterogeneity was substantial (*I*^2^ > 75%); (c) Imprecision: Certainty was downgraded by one level when results were not statistically significant; (d) Publication bias: In line with the Cochrane Handbook, formal assessment of publication bias (e.g., Egger’s test) was not performed due to the limited number of included studies (<10). Consequently, no downgrading was applied for this domain. However, the small number of studies reduces the statistical power to detect potential publication bias; therefore, this should be interpreted as a limitation of the review rather than evidence of absence (Supplementary Table 4).

Given the limited and inconsistent control of menstrual cycle phase, along with insufficient reporting of oral contraceptive use across studies, these factors were prespecified as potential sources of physiological heterogeneity. They were therefore considered during qualitative synthesis and interpretation. This limitation also informed a more cautious appraisal of the overall certainty of evidence.

### 2.6 Statistical analysis

Data synthesis was conducted using the inverse-variance weighting method within a random-effects framework, with model parameters estimated using the DerSimonian–Laird method (55). Between-study variance (*τ*^2^ and *τ*) and their corresponding confidence intervals were estimated using the Jackson method (55). Given that some included studies reported multiple non-independent effect sizes (e.g., different performance outcomes, physiological variables, or multiple comparisons within the same sample), a three-level meta-analytic model was employed to account for the dependency among effect sizes and to reflect the hierarchical structure of the data (56, 57). In this model, effect sizes were nested within studies, allowing the total variance to be partitioned into sampling variance (level 1), within-study variance (level 2), and between-study variance (level 3). This approach enables the inclusion of all eligible outcomes while appropriately accounting for within-study dependencies, thereby avoiding the loss of information and conservative bias associated with selecting a single effect size or averaging across outcomes. Because outcome measures varied in scale across studies, pooled effect sizes were expressed as standardized mean differences (SMDs) with corresponding 95% confidence intervals (95% CIs). Given the generally small sample sizes of the included studies, Hedges’ *g*, which corrects for small-sample bias, was used as the primary effect size estimate (hereafter referred to as ES). Effect sizes were interpreted according to established thresholds: <0.2 as trivial, 0.2–0.5 as small, 0.5–0.8 as moderate, and >0.8 as large (58).

Heterogeneity was assessed by calculating 95% confidence intervals (CI) and using multiple statistical metrics, including Cochran’s *I*^2^ statistic and Tau^2^. To more comprehensively capture the potential variability expected in future similar studies, prediction intervals (PI) were additionally computed, and all indicators were reported concurrently (59). In accordance with recommendations from prior methodological studies, the magnitude of heterogeneity was primarily interpreted based on *I*^2^ values, categorized as follows: 0–25% indicating low heterogeneity, 25–75% indicating moderate heterogeneity, and >75% indicating high heterogeneity (60). Subgroup analyses (61) were pre-specified to explore potential sources of heterogeneity when substantial between-study heterogeneity was detected (*I*^2^ > 50%). These analyses were conducted based on *a priori* defined study characteristics when sufficient data were available.

Leave-one-out sensitivity analyses were conducted for outcomes with three or more studies by sequentially omitting one study and recalculating the pooled effect. Analyses were not performed for outcomes with only two studies, as omitting one study would preclude recalculation of a pooled estimate (62). Sensitivity findings were summarized narratively and considered in the interpretation of the results.

All statistical analyses and graphical visualizations were conducted using R (version 4.5.0; RStudio, Inc., Boston, MA, USA) with the meta and metafor packages. Statistical significance was set at *p* < 0.05, while *p*-values between 0.05 and 0.10 were interpreted as indicating a statistical trend.

## 3 Results

### 3.1 Literature screening process

**Figure 1:**
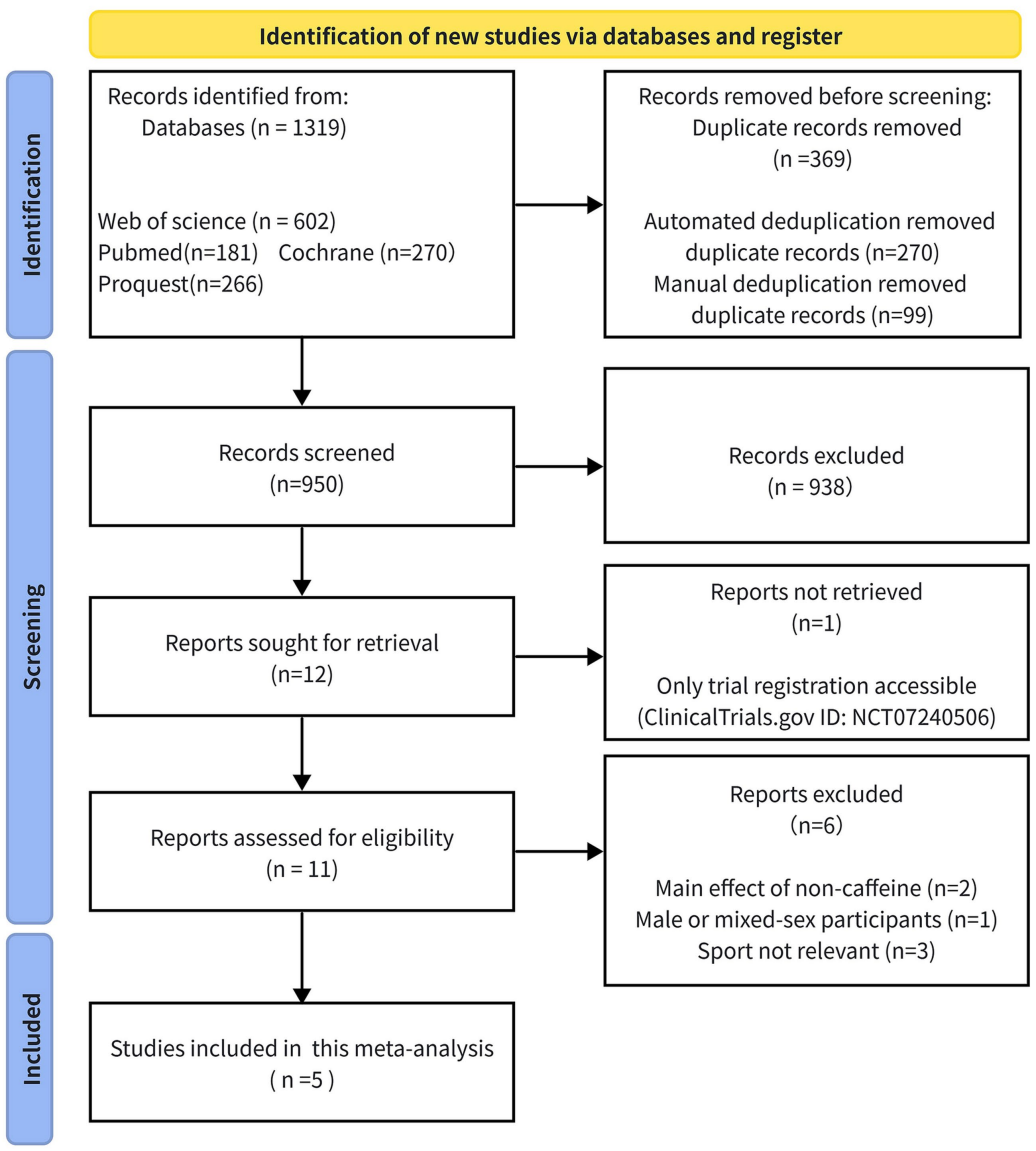
PRISMA flow diagram showing study selection.

A systematic search of five electronic databases, including PubMed (*n* = 181), Web of Science (*n* = 602), ProQuest (*n* = 266), and the Cochrane Library (*n* = 270), yielded a total of 1,319 records. After removing duplicates and screening for eligibility, five studies met the inclusion criteria (32, 36, 37, 39, 40) (Figure 1), comprising 66 female volleyball players. In addition, the reference lists of all included studies were examined, but no additional studies were identified.

As shown in Table 1, the general characteristics of the included studies comprised the following: (a) author, year of publication, and country; (b) study design; (c) participant characteristics (sample size, as well as reported training status, age, body mass, height, and menstrual cycle phase); (d) caffeine intake or restriction status; (e) intervention and comparator details (form, timing, and dosage); (f) study duration and washout period; (g) performance and physiological outcome measures; and (h) funding sources.

**Table 1:** Characteristics of included studies.

Author(s)/year/country	Study design	Participants’ characteristics	Caffeine consumption or restrictions	Menstrual cycle/Oral contraceptive	Intervention (form, dosage, and timing)	Comparator(s) (form, dosage, and protocol)	Study period and washout	Performance tests	Funding source
Author: Pérez et al. (40) Year:2015 Country: Spain	Randomized, double-blind, crossover design	*N* = 13 Age = 25.2 ± 4.8 y BM = 64.4 ± 7.6 kg Height = 174 ± 9 cm Training level = Second division of the Spanish National league (experience of at least 6 Y)	Abstinence required	Menstrual Cycle: Follicular phase (*n* = 4) and Luteal phase (*n* = 9) Oral Contraceptive: Not specified	Constituents: Caffeine Form: Energy drink Dosage: 3 mg/kg Timing: 60 min pre-test	Constituents: Maltodextrin Form: Same volume Dosage: 6.6 mg/kg Timing: Same time	1 week apart	• CMJ/SJ (↑*) • Spike/Block jump (↑*) • R/L Handgrip force (↑*) • SJ Peak power (↑*) • Block jump Peak power (↑*) • Spike ball performance (↑*) • Agility t-test (↑*) • Game actions (=)	NR
Author: Fernández et al. (32) Year: 2015 Country: USA	Randomized, double-blind, crossover design	*N* = 19 Age = 22.3 ± 4.9 y BM = 65.2 ± 10.1 kg Height = 171.8 ± 9.4 cm Training level = Years in the elite league (8.9 ± 4.5 Y)	Not restricted	Not specified	Constituents: A mixed formulation with caffeine as the primary active ingredient. Form: Energy Drink Dosage: 2 mg/kg Timing: 30 min pre-test	Constituents: Carbonated water and sugar free artificial flavors Form: Same scheme Dosage: Same volume Timing: Same time	1 week apart	• R/L Hand grip strength (↑*) • CMJ/SJ (↑) • Peak/Mean power (=)	NR
Author: Filip-Stachnik et al. (a) (37) Year: 2022 Country: Poland	Randomized, double-blind, crossover design	*N* = 14 Age = 26 ± 3 y BM = 62.6 ± 5.6 kg Height = 171 ± 5 cm Training level = Least 7 years of volleyball training (13 ± 3 Y)	2.9 ± 2.4 mg/kg/b.m/day	Not specified	Constituents: Caffeine Form: Capsule Dosage: 6 mg/kg Timing: 60 min pre-test	Constituents: All-purpose flour Form: Capsule Dosage: Same volume Timing: Same time	Least 72 h	• Repeat CMJ (↑)	Ministry of Science and Higher Education under the 2019–2022 Regional Initiative of Excellence program, project number: 022/RID/2018/19, grant amount: PLN 11,919,908.
Author: Filip-Stachnik et al. (b) (36) Year: 2022 Country: Poland	Randomized, crossover design	*N* = 12 Age = 20 ± 2 y BM = 69.1 ± 2.3 kg Height = 178 ± 6 cm Training level = 10 ± 2 Y	2.7 ± 2.1 mg/day	Menstrual Cycle: Follicular phase (*n* = 6) and Luteal phase (*n* = 6) Oral Contraceptive: Not specified	Constituents: Caffeine Form: Gum Dosage: ~6.4 mg/kg Timing: 15 min pre-test	Constituents: Inert substance (caffeine-free); exact composition not reported Form: Gum Dosage: Same volume Timing: Same time	72 h	• Attack Jump (↑*) • Block Jump (↑) • Game assessment • Side effects	Ministry of Science and Higher Education under the 2019–2022 Regional Initiative of Excellence program, project number: 022/RID/2018/19, grant amount: PLN 11,919,908
Author: Siquier et al. (39) Year: 2023 Country: Spain	Randomized, double-blind, crossover design	*N* = 8 Age = 17–25 y BM = 66.67 ± 4.74 kg Height = 1.63 ± 0.08 m Training level = Semi-professional (least 5 Y of experience)	Lower than 100 mg/day	Menstrual Cycle: (luteal/follicular), *n*: supplementation 5/3; placebo 4/4 Oral Contraceptive: No medication was taken within the past 6 month.	Constituents: Caffeine (Mixed with a maltodextrin) Form: Beverage Dosage: 5 mg/kg Timing: 60 min pre-test	Constituents: Only maltodextrin Form: Beverage Dosage: Same volume Timing: Same time	1 week apart	• CMJ (↑*) • Handgrip dominant (↑*) • Handgrip non-dominant (↑) • COD 5050 test (=) • Yo-Yo test (=)	NR

*N* = total number of participants; Y = year; BM = body mass; N/A = not available; NR = not reported; CMJ = countermovement jump; SJ = squat jump; R = right; L = left; COD 5050 test = chante of direction 505 test; (↑) = improvement or enhancement of the effect; (=) = no noticeable change in effect; (↓) = reduction or decline of the effect; (*) = statistically significant effect (*p* < 0.05).

### 3.2 Risk of bias assessment in included studies

According to the PEDro scale, the methodological quality of the included studies ranged from 7 to 9, indicating an overall high level of quality. The PEDro scores were distributed as follows: two studies achieved a score of 9 (36, 40), one study scored 8 (37), and two studies scored 7 (32, 39). All studies met key methodological criteria, including random allocation, baseline comparability, between-group comparisons, and the reporting of point estimates and measures of variability. However, several methodological limitations were identified. Four studies (32, 36, 37, 39) did not clearly satisfy the requirement for assessor blinding, and three studies (32, 37, 39) did not clearly meet the criterion for therapist blinding. In addition, three studies (32, 36, 40) did not fully meet the criteria related to intention-to-treat analysis or completeness of follow-up. Furthermore, only one study (39) did not clearly report allocation concealment. Despite the overall high methodological quality, these limitations resulted in moderate concerns in specific PEDro item (Supplementary Table 2).

According to the RoB 2 assessment (Supplementary Table 3), all five included studies were judged to have some concerns of bias, with none rated as having a high risk of bias. At the domain level, all studies were assessed as having some concerns in Domain 5 (selection of the reported result), as none explicitly reported trial registration information or a pre-specified statistical analysis plan. In addition, two studies (32, 36) were judged to have some concerns in Domain 3 due to incomplete outcome data and insufficient information to determine whether the missing data could have introduced bias. Furthermore, one study (39) was judged to have some concerns in Domain 1 because the method used for random sequence generation was not adequately reported.

**Figure 2:**
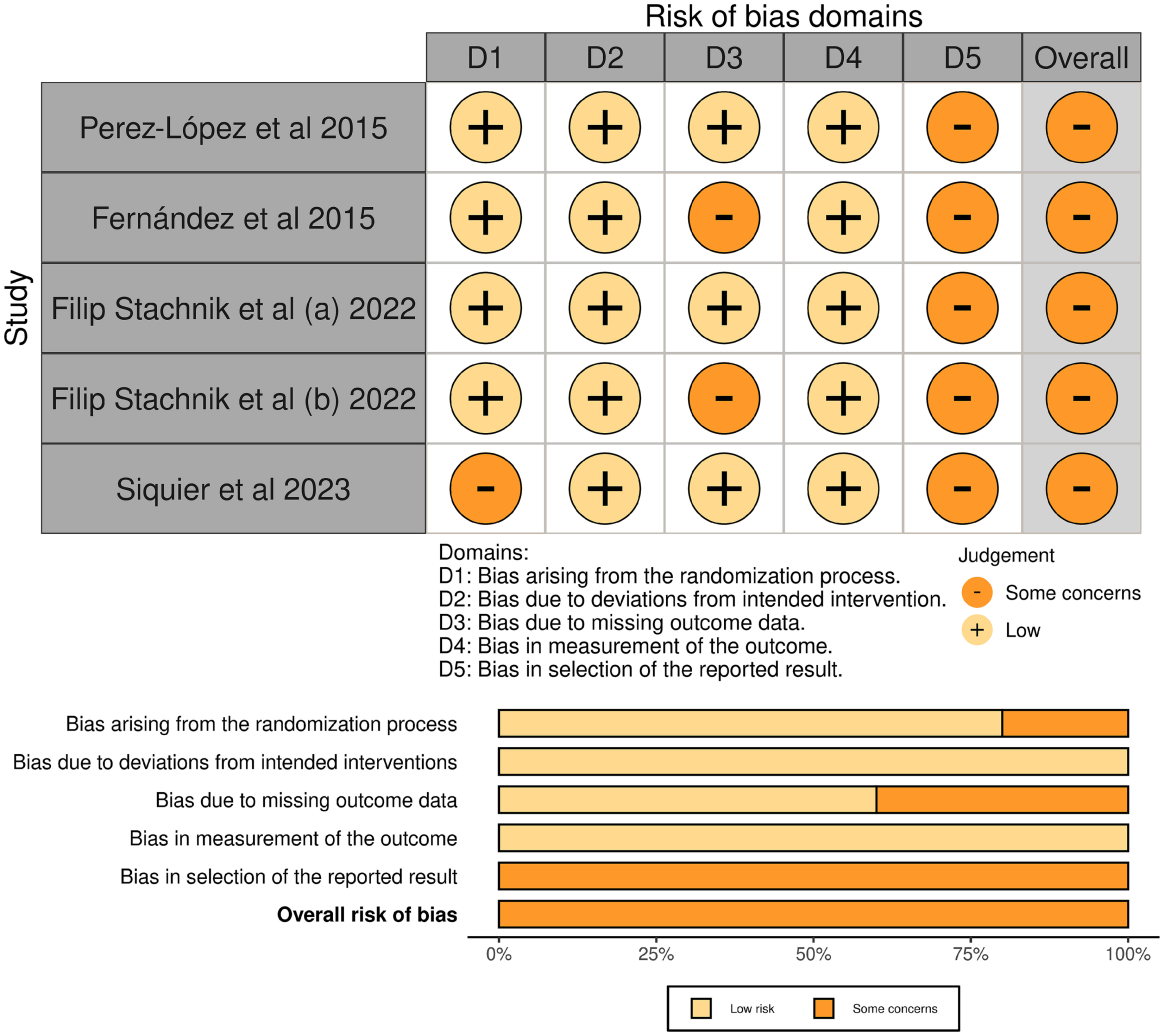
Risk of bias results.

Based on the PEDro and RoB 2 evaluations, a risk of bias plot was generated using the Robvis tool (63) (Figure 2).

### 3.3 Participants and studies characteristics

A total of five randomized controlled studies involving 66 female volleyball players were included (32, 36, 37, 39, 40). Sample sizes ranged from 8 to 19 participants, comprising professional, elite/high-level, and semi-professional athletes. Mean age ranged from ~20 to 26 years, with one study including a wider range (17–25 years) (39). The athletes included in the studies generally possessed substantial volleyball experience, ranging from 5 to 13 ± 3 years. Participants were classified as semi-professional, national-level, or elite competitors. Weekly training volume was reported in only two studies: Pérez-López et al. (40) documented five 2-h sessions per week, whereas Siguier et al. (39) reported three 90-min sessions plus one match per week. Other studies did not provide sufficient information regarding weekly training hours. Anthropometric characteristics were broadly comparable across studies, with body mass ranging approximately from 62 to 69 kg and height from 1.63 to 1.78 m, reflecting trained female volleyball populations. Except for one study that neither restricted nor reported habitual caffeine intake (32), all other studies included athletes characterized by low-to-moderate daily caffeine consumption.

All included studies were published between 2015 and 2024 and were conducted in European and Latin American settings, reflecting a relatively recent and geographically limited body of evidence. All trials adopted within-subject experimental designs, ensuring a consistent methodological approach across studies. Despite this general consistency, some variability existed in study context, including differences in competitive level and training environment, which may contribute to clinical heterogeneity and should be considered when interpreting the overall findings.

### 3.4 Caffeine supplementation protocol and dosage characteristics

The caffeine doses administered in the included studies ranged from 2 to 6 mg/kg. Regarding the form of administration, three studies employed energy drinks or beverage-based delivery, while one study each utilized caffeine chewing gum and capsules, respectively. In terms of timing, caffeine was administered 15–60 min prior to the commencement of the performance tests. Specifically, three studies (37, 39, 40) administered caffeine 60 min prior to testing, with one study each providing caffeine at 30 min (32) and 15 min (36) before testing. In all cases, the reported timing refers to the interval between caffeine ingestion and the start of the actual performance assessments. Concerning washout periods, three studies (32, 39, 40) implemented a one-week washout period, whereas two studies (36, 37) adopted a minimum washout duration of 72 h. Detailed information on supplementation protocols and timing is summarized in Table 1.

### 3.5 Menstrual cycle and oral contraceptives

Among the studies included in this review, three studies (36, 39, 40) reported the menstrual cycle status of participants. In these studies, all participants were tested during either the luteal or follicular phase. However, only one study (39) specified the method used to determine menstrual cycle phase, estimating it based on retrospective self-reports of the timing of the last menstrual period, while the remaining two studies did not clearly describe how cycle phase was determined. Two studies (32, 37) did not report menstrual cycle phase, nor did they indicate whether experimental scheduling was adjusted according to cycle phase. Regarding control of oral contraceptive use, only one study (39) explicitly required participants to have abstained from any medication use in the preceding 6 months (which may have implicitly restricted oral contraceptive use). In contrast, the other four studies neither reported participants’ oral contraceptive use nor specified whether this factor was controlled. Further details are provided in Table 1.

### 3.6 Meta-analysis results

In Sections 3.6.1–3.6.3, the combined sample size refers to the total number of participant-observations contributing to each meta-analysis outcome. When a single study reported multiple eligible outcomes or testing conditions within the same domain, these were included as separate effect sizes. Accordingly, the number of effect sizes may exceed the number of studies, and combined sample sizes may differ across outcomes.

#### 3.6.1 Spiking performance

Two studies (36, 40) (comprising six effect sizes; total sample size = 150) were included. Outcomes assessed included spike velocity, spike jump height, and attacking jump performance (including jump height, scoring, and errors). The meta-analysis showed no significant effect of caffeine intake on the offensive performance of female volleyball players (ES = 0.17, 95% CI: −0.43 to 0.75; *p* = 0.504). Heterogeneity was low (*I*^2^ = 0%, *τ*^2^ = 0.000).

#### 3.6.2 Physical performance

##### 3.6.2.1 Jump height performance

Four studies (32, 37, 39, 40) (comprising 22 effect sizes; total sample size = 490) were included. Outcomes assessed included countermovement jump (CMJ), squat jump (SJ), and repeated jump tests that better reflect the characteristics of volleyball competition (jump height). The meta-analysis indicated that caffeine intake did not have a significant effect on jump performance of female volleyball players (ES = 0.16; 95% CI: −0.43 to 0.17; *p* = 0.505). Heterogeneity was low (*I*^2^ = 0%, *τ*^2^ = 0.000).

##### 3.6.2.2 Change-of-direction performance (agility)

Two studies (39, 40) (comprising 4 effect sizes; total sample size = 74) were included. Outcomes assessed included the T-test and the 505 test. The meta-analysis showed no significant effect of caffeine intake on agility performance in female volleyball players (ES = 0.26; 95% CI: −1.16 to 0.65; *p* = 0.432). Heterogeneity was low (*I*^2^ = 0%, *τ*^2^ = 0.000).

##### 3.6.2.3 Handgrip strength performance

Three studies (32, 39, 40) (comprising 10 effect sizes; total sample size = 224) were included. Outcomes assessed included handgrip strength measurements of the left and right hands, or the dominant and non-dominant hands. The meta-analysis indicated that caffeine intake did not have a statistically significant effect on handgrip strength in female volleyball players (ES = 0.29; 95% CI: −0.38 to 0.95; *p* = 0.352). Moderate heterogeneity was observed (*I*^2^ = 44.4%, *τ*^2^ = 0.149).

##### 3.6.2.4 Power output

Two studies (32, 40) (comprising 5 effect sizes; total sample size = 154) were included. Outcomes assessed included peak or mean power during jumping, as well as peak or mean power measured during physical performance tests. The meta-analysis showed no significant effect of caffeine intake on power output in female volleyball players (ES = 0.16; 95% CI: −0.43 to 0.74; *p* = 0.505). Heterogeneity was low (*I*^2^ = 0%, *τ*^2^ = 0.000).

#### 3.6.3 Physiological reactions

##### 3.6.3.1 Fatigue-related outcomes

Three studies (32, 39, 40) (comprising eight effect sizes; total sample size = 160) were included. Outcomes assessed included ratings of perceived fatigue and fatigue index. The meta-analysis showed no significant effect of caffeine intake on fatigue-related outcomes in female volleyball players (ES = 0.09; 95% CI: −0.59 to 0.78; *p* = 0.757). Moderate heterogeneity was observed (*I*^2^ = 46.4%, *τ*^2^ = 0.183).

##### 3.6.3.2 Heart Rate

Two studies (36, 40) (comprising four effect sizes; total sample size = 100) were included. Outcomes assessed included maximal heart rate and mean heart rate. The meta-analysis showed no significant effect of caffeine intake on heart rate–related outcomes in female volleyball players (ES = 0.33; 95% CI: −0.70 to 1.36; *p* = 0.382). Moderate heterogeneity was observed (*I*^2^ = 34.4%, *τ*^2^ = 0.086).

### 3.7 Potential adverse effects

Only two studies explicitly reported adverse events occurring within 24 h following caffeine ingestion (see Table 2). Pérez et al. (40) reported occurrences of nervousness and activeness based on self-report questionnaires, with incidence rates of 31% vs. 0 and 15% vs. 0%, respectively, compared with the placebo group. Additionally, Filip-Stachnik et al. (36) reported one case of hand tremor based on post-exercise self-report corresponding to an adverse events of 8.3% based on the total sample size (0% in the placebo group). It is worth noting that the reported potential adverse effects may be associated with enhanced central nervous system activation induced by caffeine intake. This phenomenon is conceptually illustrated as “excessive stimulation” in the graphical abstract, representing a general depiction of central nervous system arousal rather than a directly measured variable.

**Table 2:** Summary of caffeine-related adverse effects.

ID	First author	Side effects	Number of participants	CAF (%)	PLA (%)
1	Pérez-López et al. 2015	Nervousness	13	31	0
2	Pérez-López et al. 2015	Activeness	13	15	0
3	Filip-Stachnik et al. 2022 (b)	Hand tremors	12	8.3	0

## 4 Discussion

### 4.1 Evidence summary

This systematic review included five studies (32, 36, 37, 39, 40) involving a total of 66 female volleyball athletes. It aimed to evaluate the acute effects of caffeine ingestion on sport-specific skills, physical performance, and physiological and biochemical outcomes, as well as to explore the potential moderating roles of caffeine dose and test duration. The meta-analysis showed no statistically significant effects across all outcomes. The overall certainty of evidence was low, partly due to potential selective reporting bias. RoB 2 assessment indicated that five studies had some concerns in Domain 5, and all studies were judged to have an overall risk of bias, suggesting limited transparency in prespecified outcomes or analytical plans. This may have increased the likelihood of selective reporting, potentially overestimating the intervention effects. Accordingly, the certainty of evidence was downgraded based on GRADE, and the pooled estimates should be interpreted with caution. Subgroup analyses were prespecified for cases of substantial heterogeneity (*I*^2^ > 50%); however, statistical heterogeneity appeared low to moderate across the analyses. Therefore, no subgroup analyses were conducted. Importantly, however, heterogeneity statistics should not be overinterpreted in the present review, because several analyses were based on a very small number of studies and effect sizes. Under these conditions, estimates of heterogeneity, variance components, and pooled effects may be unstable. Given the small number of studies and limited sample size, further subgroup analyses may increase the risk of spurious findings and overinterpretation.

Although clinical and methodological heterogeneity existed across the included studies, all studies examined the acute effects of caffeine ingestion in female volleyball players and assessed broadly related performance or physiological outcomes. Therefore, outcomes were grouped into predefined domains and synthesized using three-level random-effects meta-analytic models to account for the clustering of multiple effect sizes within the same studies or participant samples. The rationale for using a three-level model was to address the non-independence of effect sizes by partitioning variance into sampling error, within-study variability, and between-study variability. Importantly, the use of this model should not be interpreted as implying a stronger level of evidential certainty. Rather, it was adopted to reduce the risk of bias that could arise from either selecting a single effect size from each study or treating multiple effect sizes from the same sample as statistically independent observations.

Nevertheless, the quantitative synthesis should be regarded as exploratory and complementary to the structured narrative synthesis, rather than as a confirmatory analysis. The included evidence base was extremely limited, comprising only five studies and 66 participants; several pooled analyses were based on as few as two studies; and multiple effect sizes were derived from the same underlying participant samples. Thus, although the three-level model was methodologically appropriate for handling dependent effect sizes, it cannot overcome the restricted information available for estimating variance components and pooled effects. Furthermore, some outcomes grouped within broad performance domains were conceptually related but not fully interchangeable. Therefore, the pooled estimates should be interpreted as approximate domain-level summaries of the overall direction and magnitude of effects, rather than as precise estimates for any single volleyball-specific performance outcome, and should be considered in conjunction with the narrative synthesis of study-specific characteristics and findings.

### 4.2 Acute effects of caffeine intake on spike performance in female volleyball players

Spiking, as one of the most critical offensive skills in volleyball, plays a decisive role in match outcomes (64). However, the present meta-analysis indicates that caffeine ingestion only demonstrates a trivial and non-significant positive effect on spiking or overall offensive performance in female volleyball players. Notably, inconsistent findings have been reported across both male and female populations regarding the effects of caffeine on volleyball-specific technical performance. These discrepancies may be largely attributed to the inherent complexity of the task. Spiking requires the integration of multiple performance determinants, including precise jump timing, spatial positioning, and trunk stability control (65–67). Such abilities are highly dependent on individual skill level and long-term training adaptations, and are difficult to standardize across studies, thereby increasing variability in outcomes (18). From a mechanistic perspective, caffeine may enhance performance via increased catecholamine release, elevated adrenaline levels, and heightened central nervous system excitability, leading to improved alertness and neuromuscular activation (68–70). Additionally, estrogen may exert a synergistic influence on these processes, further supporting the maintenance of an activated physiological state (71). These effects may provide a physiological basis for potential improvements in offensive performance.

Nevertheless, perceptual–motor abilities such as spatial awareness and timing are primarily developed through long-term sport-specific training and thus are unlikely to be substantially improved through caffeine ingestion alone. This is consistent with findings from Zhang et al. (18) in female basketball players, where caffeine supplementation did not produce meaningful enhancements in sport-specific technical performance. However, in athletes with already high levels of technical proficiency, the physiological effects of caffeine may interact with existing skills, resulting in small but potentially meaningful performance gains. Such marginal benefits may be more evident in high-level female volleyball players.

### 4.3 Acute effects of caffeine intake on physical performance in female volleyball players

This meta-analysis did not reveal a statistically significant effect of caffeine ingestion on jump height in female volleyball players, and the magnitude of the effect suggests only limited practical benefit. Nevertheless, enhancing jumping ability remains a central focus in volleyball research and practice, as it is a key determinant of high-level performance and competitive success. Recent volleyball-specific meta-analyses have reported that acute caffeine ingestion improves overall jumping performance in volleyball players, including single- and repeated-jump outcomes, although its effects on volleyball-specific attack and block jump height appear less consistent (41, 42). The included studies in the present analysis also showed inconsistent findings. Filip Stachnik et al. (37) reported that a single dose of 6 mg/kg caffeine did not improve jump performance in female athletes, whereas Pérez et al. (40) and Siquier et al. (39) reported improved jump-related outcomes following approximately 3 and 5 mg/kg caffeine, respectively. These findings are consistent with the general trend observed in studies involving male volleyball players (33). A plausible explanation for this discrepancy is the lack of control for menstrual cycle phase in the study by Filip Stachnik et al. (37) Fluctuations in estrogen across the menstrual cycle may modulate the ergogenic effects of caffeine, leading to variability in response magnitude across different phases. Recent evidence suggests that the effects of caffeine on jumping performance may be relatively more pronounced during the follicular phase (72). From a mechanistic perspective, caffeine may enhance muscle contractility by increasing Na^+^/K^+^-ATPase activity and promoting Ca^2+^ release, thereby providing a physiological basis for improvements in lower-limb explosive power and jumping performance (24, 73). In addition, higher estrogen levels in females may further potentiate or prolong these effects (74). Coupled with a relatively greater proportion of type I muscle fibers (75, 76), female athletes may, under certain conditions, exhibit a comparable or even greater potential to benefit from caffeine than males. However, it should be noted that the current evidence remains limited, and these observations do not support specific practical recommendations, including caffeine use across different phases of the menstrual cycle. Therefore, this interpretation should be considered as a potential explanatory mechanism and a direction for future research rather than a basis for applied practice.

In the present meta-analysis, caffeine ingestion did not exert a statistically significant effect on agility performance in female volleyball players. This finding is consistent with previous evidence in female cohorts, which similarly failed to demonstrate a significant ergogenic effect on agility-related outcomes (18, 48). Nevertheless, recent volleyball-specific meta-analyses suggest potential benefits of caffeine for agility or running-based performance in mixed-sex samples, particularly with ingestion ~60 min before exercise (41, 42). However, it is noteworthy that the two studies (39, 40) included in this analysis reported discrepant findings, suggesting potential methodological and inter-individual sources of heterogeneity. One plausible explanation relates to differences in testing protocols. Specifically, the T-test and the 505 change of direction test (COD 505) impose distinct demands on cognitive processing, directional change mechanics, and lower limb power (77, 78), which may differentially influence responsiveness to caffeine. In addition, inter-individual variability, particularly genetic polymorphisms and habitual caffeine intake, may have attenuated the pooled effect. Evidence indicates that both genotype and habitual consumption modulate the ergogenic response to caffeine, contributing to substantial between-subject variability and potentially obscuring small but meaningful effects at the group level (79–81). Despite the absence of a significant overall effect, mechanistic evidence suggests that caffeine enhances central nervous system drive, reduces reaction time, and increases motor unit recruitment. These effects provide a plausible physiological basis for potential improvements in agility performance, although such benefits may be context dependent and not consistently detectable under current testing paradigms (27, 82). Thus, any potential caffeine-related benefit for agility in female volleyball players may be context dependent and difficult to detect consistently under heterogeneous testing protocols.

In the present meta-analysis, caffeine ingestion did not produce a statistically significant effect on handgrip strength in female volleyball players. Handgrip strength is widely used as a convenient surrogate of upper limb muscular strength; however, as it primarily reflects hand and forearm flexor function, its ability to represent multi-joint upper limb strength remains limited and should be interpreted with caution. Findings from the three included studies were inconsistent. Pérez et al. (40) and Siquier et al. (39) reported significant improvements following 3 to 5 mg per kg of caffeine, although no effect was observed in the non-dominant hand in the latter. These findings are broadly consistent with recent volleyball-specific meta-analyses reporting small but significant benefits of caffeine on strength-based performance, including handgrip strength (41, 42). In contrast, Fernández et al. (32) reported no significant effect at a lower dose of 2 mg per kg. Such discrepancies may be attributed to differences in caffeine dose and mode of administration. A dose of 2 mg per kg may be insufficient to elicit detectable effects, whereas approximately 3 mg per kg may represent a threshold (32, 40, 83), with potential variability across the range of 3 to 6 mg/kg (18). In addition, all studies administered caffeine via energy drinks, in which carbohydrates and other constituents may influence absorption kinetics, energy availability, or perceptual responses, thereby contributing to variability in outcomes. This methodological heterogeneity should be acknowledged when interpreting the findings. However, despite these differences, all interventions involved caffeine ingestion as the primary ergogenic component, allowing for a meaningful quantitative synthesis. Furthermore, the use of a random-effects model accounted for between-study variability, providing a more conservative estimate of the overall effect. Nevertheless, the results should be interpreted with caution, and future studies employing standardized caffeine administration protocols are warranted to improve comparability.

The present study showed that caffeine ingestion did not significantly improve power output in female volleyball players. Although the direction of effect was positive, the confidence interval crossed zero, indicating a lack of statistical robustness. This finding may partly stem from heterogeneity in outcome constructs. Fernández et al. (32) employed the Wingate test to assess anaerobic power, whereas Pérez-López et al. (40) evaluated explosive power through jump-based tasks. These two approaches differ fundamentally in movement patterns and muscle recruitment characteristics, which may have attenuated the detectability of pooled effects. Moreover, the inconsistent directional findings between these studies further underscore the influence of testing paradigms on outcomes. Additionally, prior research indicates that caffeine-induced improvements in power output are primarily evident during repeated jumping tasks involving accumulated fatigue (84–86). However, its effects on power output during single maximal-effort jumps remain equivocal, and how this discrepancy manifests in volleyball-specific contexts warrants further investigation (33, 84, 87). From a physiological perspective, evidence suggests that caffeine reduces pain perception and suppresses both excessive and perceived fatigue (39, 88, 89). Combined with its capacity to promote adrenaline release, increase lactate production, and modulate acetylcholine and dopamine secretion, these pharmacological effects may theoretically delay fatigue development (39, 90) and thereby help athletes maintain or enhance performance during repeated jumping tasks. Thus, caffeine may be more likely to support performance maintenance under fatigue than to increase peak power in isolated maximal tests.

### 4.4 Acute effects of caffeine intake on physiological reactions in female volleyball players

This meta-analysis found no statistically significant effect of caffeine ingestion in female volleyball players, a result broadly consistent with previous evidence showing limited or inconsistent anti-fatigue effects of caffeine. Although caffeine may reduce perceived fatigue through adenosine receptor antagonism, increased central nervous system excitability, and elevated *β*-endorphin levels (91, 92), these potential benefits may be constrained in the context of volleyball. Volleyball is a typical high-intensity intermittent sport characterized by the rapid accumulation of both central and peripheral fatigue, together with brief intervals of recovery. Under such repeated high-frequency, high-intensity demands, fatigue is likely driven by multiple factors, and a centrally mediated stimulatory effect alone may be insufficient to meaningfully alter overall fatigue status. Therefore, the movement demands and competitive context of volleyball may partly attenuate the practical effect of caffeine on fatigue.

The present meta-analysis demonstrated that acute caffeine ingestion did not produce statistically significant effects on heart rate parameters (including maximal heart rate and mean heart rate) during exercise in female volleyball players. Although caffeine may theoretically elevate heart rate through enhanced adrenaline release, the current findings are consistent with the majority of existing literature showing no significant caffeine-induced heart rate alterations in athletes (33, 93–96). It should be noted, however, that considerable heterogeneity exists within the existing literature, with some studies reporting positive effects of caffeine on athletic performance (97–100). Regarding the null findings of the present study, one plausible explanation is that the included athletes had training histories of ≥6 years, and chronic systematic training may induce adaptive cardiovascular remodeling (e.g., athlete’s heart phenotype), thereby attenuating the sensitivity of heart rate responses to exogenous caffeine. Notably, emerging evidence suggests that the cardiovascular effects of caffeine may be modulated by genetic polymorphisms, with CYP1A2 CC slow-metabolizer genotype individuals potentially exhibiting heightened susceptibility to caffeine-induced adverse effects such as elevated heart rate (101). Consequently, in intermittent high-intensity sports such as volleyball, the potential genotype-dependent adverse cardiovascular effects warrant careful consideration. Future research should specifically investigate the genotype-caffeine interaction in volleyball-specific athlete populations to further elucidate this issue.

### 4.5 Caffeine supplementation strategies

From a dosage perspective, the present study included one investigation (32) employing an ultra-low caffeine dose of approximately 2 mg/kg, which failed to produce statistically significant effects in female volleyball players. This finding aligns with the prevailing consensus that the effective acute caffeine intake range is typically 3 to 6 mg/kg (69), with approximately 3 mg/kg commonly regarded as the threshold dose for ergogenic effects, a conclusion that has been substantiated in several volleyball-specific studies (32, 40, 41). Notably, however, emerging evidence has documented potential ergogenic effects of ultra-low caffeine doses (below 3 mg/kg) on certain performance metrics (108, 109), suggesting that the dose–response relationship of caffeine may exhibit context dependency, whereby lower doses may still confer beneficial effects under different exercise modalities. Nevertheless, based on current evidence, ultra-low dose caffeine supplementation appears insufficient to elicit significant ergogenic effects in intermittent high-intensity sports such as volleyball or basketball.

From the perspective of ingestion form and timing, both are important factors influencing the effectiveness of caffeine interventions (102). Among the studies included in this review, two studies (32, 40) employed energy drinks formulations, while the others used capsules (37), beverages (39), and chewing gum (36) respectively. Current evidence suggests that caffeine in capsule form is typically ingested approximately 60 min prior to exercise to ensure that plasma caffeine concentrations approach peak levels at the onset of activity (103). Energy drinks or standard beverages are generally consumed 30 or 60 min before exercise (32, 40, 84). In contrast, caffeine-containing chewing gum allows for more rapid absorption via the buccal mucosa, reaching peak plasma concentrations within a shorter time frame; thus, ingestion approximately 15 min before exercise is usually sufficient (104). This feature provides practical advantages in situations where pre-exercise preparation time is limited. However, it should be noted that energy drinks often contain additional active ingredients, such as taurine and sodium bicarbonate, which may exert synergistic or interfering effects on exercise performance. Consequently, these components may either enhance or attenuate the effects of caffeine itself, and the results of such studies should therefore be interpreted with caution. Furthermore, from the perspective of supplement preparation and dosage control, energy drinks and chewing gum (105) present challenges in achieving precise dosing during actual consumption, whereas capsule formulations offer more substantial advantages in terms of dose standardization and controllability.

**Figure 3:**
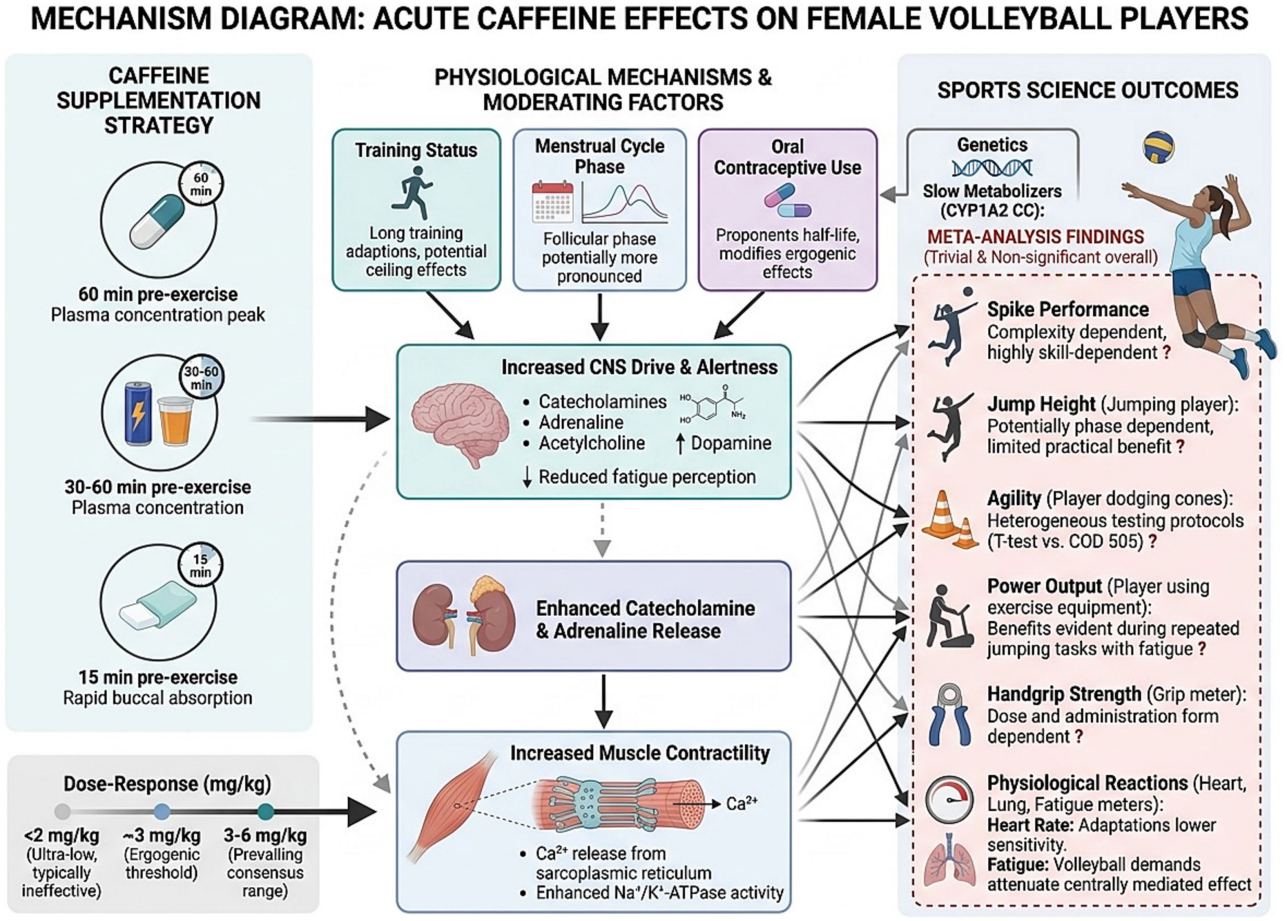
Proposed mechanisms underlying the potential effects of caffeine on performance in female volleyball players. This figure summarizes plausible mechanisms proposed in the existing literature. These pathways should be interpreted as hypothesis-generating rather than as confirmed explanations of the present meta-analysis findings, as the pooled outcomes did not reach statistical significance.

Figure 3: The observed outcomes did not show statistically significant effects in this study. Therefore, these findings should be interpreted with caution. The related discussions are informed by existing literature and remain speculative.

### 4.6 Menstrual cycle phase, training status and oral contraceptive use

Training status, menstrual cycle phase, and oral contraceptive use are key moderating variables that may contribute to the heterogeneity observed in female-specific exercise enhancement responses. It has been suggested that training status may influence the magnitude of caffeine’s ergogenic effects, with well-trained athletes typically demonstrating smaller relative improvements in exercise performance due to neuromuscular adaptations and ceiling effects, whereas individuals with lower training levels may exhibit greater variability (18). Consequently, differences in volleyball training experience and neuromuscular training status may modulate the magnitude of exercise performance changes following caffeine or energy drink ingestion.

Hormonal fluctuations during the menstrual cycle may further influence metabolic and neuromuscular responses, including substrate utilization, thermoregulation, and fatigue perception (106, 107). Although Perez et al. (40) suggested that the acute exercise-enhancing effects of caffeine may be generally comparable between the follicular and luteal phases, this study acknowledges that interindividual variability remains substantial, and subtle phase-dependent differences cannot be entirely ruled out. Therefore, the lack of control over or failure to report menstrual cycle information in studies may introduce additional sources of variability, subsequently contributing to inconsistencies in research findings. The use of oral contraceptives is another potential factor that may influence ergogenic effects. As exogenous hormones, oral contraceptives (45) can alter caffeine metabolism by modulating cytochrome P450 (CYP1A2) activity, potentially prolonging its half-life and thereby affecting its physiological effects (79).

In summary, these factors highlight the importance of considering training status, menstrual cycle phase, and oral contraceptive use in future research. The interactions among these variables are likely to influence the ergogenic responses in female athletes and should be carefully addressed to improve the reproducibility of findings and to support more individualized nutritional strategies.

### 4.7 Sensitivity analyses

The leave-one-out sensitivity analyses (Supplementary Table 5) suggested that the main findings were not materially driven by any single study. For jump height performance, sequential omission of each study did not alter the overall non-significant effect, and heterogeneity remained low. Notably, when Filip-Stachnik et al. was omitted, the pooled effect approached statistical significance, but the confidence interval still crossed zero, indicating that the conclusion remained unchanged.

For handgrip strength performance, removal of individual studies resulted in some variation in the magnitude of the pooled effect and in *I*^2^ values; however, all recalculated estimates remained statistically non-significant. Therefore, the overall interpretation of no significant pooled effect was not materially affected by any single study.

For fatigue-related outcomes, the pooled effect also remained non-significant after sequential omission of individual studies. Although the direction of the effect changed slightly when Pérez-López et al. was removed, the effect estimate remained close to zero and statistically non-significant. In addition, the estimate became highly imprecise when Siguier et al. was omitted, suggesting that this outcome should be interpreted cautiously because of the limited number of available studies and wide uncertainty around the pooled estimate.

Overall, the sensitivity analyses indicated that the main conclusions were not driven by a single study. However, these analyses should not be interpreted as strong evidence of robustness, because the number of included studies and participants was very small, and several outcome domains contained only a limited number of effect sizes. Therefore, the apparent stability of the pooled estimates should be viewed cautiously and regarded as preliminary rather than confirmatory. Further high-quality studies in female athletes are needed to confirm these results.

### 4.8 Study limitations

This meta-analysis has several limitations. First, only five studies (*n* = 66) were included, which substantially limits the precision, robustness, and generalizability of the pooled estimates and warrants cautious interpretation, reflecting the still limited evidence in female volleyball players. Several pooled analyses were based on as few as two studies, and multiple effect sizes were derived from the same underlying participant samples. Although three-level meta-analytic models allowed the dependence among effect sizes to be modelled more appropriately, they cannot compensate for the limited evidence base or eliminate uncertainty in the estimation of variance components, heterogeneity statistics, and overall pooled effects. Second, two included studies (32, 40) used energy drinks as the caffeine source, introducing potential confounding effects from additional ingredients (e.g., taurine, carbohydrates), thereby limiting causal inference regarding the independent effect of caffeine. Furthermore, methodological and clinical heterogeneity existed across studies, including differences in caffeine forms, dosages, timing of administration, training status, and outcome assessment protocols. Although outcomes were grouped into predefined, broadly related domains and statistical heterogeneity appeared generally low to moderate, some measures within these domains were conceptually related but not fully interchangeable, which may limit the interpretability and practical meaning of pooled domain-level estimates. Consequently, the results of the meta-analysis should be regarded as preliminary and exploratory rather than conclusive. Finally, although some studies reported menstrual cycle phases, these were generally based on self-report without objective hormonal verification, making it difficult to clarify the role of endocrine status in modulating caffeine effects.

Future studies should include larger and more diverse samples to improve statistical power and generalizability, use isolated caffeine interventions to distinguish the effects of caffeine from those of co-ingested ingredients, and better control female-specific variables through standardized menstrual cycle tracking and hormonal assessment. These recommendations are justified by limitations of the included studies and by recent volleyball-specific meta-analyses highlighting the need to refine caffeine dosing strategies and consider individual modifiers, including habitual caffeine intake, player characteristics, and sex-specific factors (41, 42). In addition, the effects of different ingestion timings (e.g., pre-, mid-, and post-exercise) should be explored, because the included studies used different timing protocols and current evidence remains insufficient to determine an optimal strategy for female volleyball players. Long-term interventions should also be considered to assess chronic adaptations, although current evidence is primarily based on acute caffeine ingestion and therefore does not allow conclusions regarding repeated or chronic use.

Overall, current evidence does not consistently support an ergogenic effect of caffeine on sport-specific or physical performance in female volleyball players. Given the limited and heterogeneous data, these findings should be considered preliminary and interpreted with caution. Taken together, these limitations indicate that the present findings should primarily be used to delineate the current boundaries of evidence, identify methodological priorities, and inform the design of future studies in female volleyball athletes, rather than to support definitive practical recommendations.

## 5 Conclusion

Overall, this systematic review and exploratory meta-analysis found no statistically significant effects of acute caffeine ingestion at doses of approximately 2–6.4 mg/kg on sport-specific performance (e.g., spike performance), physical capacities (e.g., jump height, change of direction, handgrip strength, and power output), or physiological outcomes (e.g., fatigue-related and psychological measures) in female volleyball players. However, these findings should not be interpreted as definitive evidence of no effect. The available evidence base is extremely limited, comprising only five studies and 66 participants, and several pooled analyses were based on very few studies and dependent effect sizes derived from the same participant samples. Therefore, the pooled estimates should be interpreted cautiously as preliminary domain-level summaries rather than robust or confirmatory estimates. Current evidence does not support a consistent ergogenic benefit of caffeine for performance enhancement in female volleyball players. Some studies also reported potential adverse effects, such as hand tremors, anxiety, or activeness. Given the small number of included studies, limited sample size, methodological heterogeneity, and uncertainty surrounding the stability of pooled estimates, further well-designed studies are required before firm conclusions or sport-specific recommendations can be made.

## Practical applications

Based on the current evidence, caffeine at doses of approximately 2–6.4 mg/kg cannot be confidently recommended as a consistently effective ergogenic strategy for improvements in sport-specific performance or physical capacities in female volleyball players. Therefore, coaches and practitioners should exercise caution when considering caffeine as an ergogenic aid in this population. Given the potential for adverse effects such as tremors, anxiety, and activeness, individualized assessment of tolerance and response is recommended, preferably conducted during training rather than in pre-competition settings, to identify an optimal individual dosing window. In practice, controlled dosing strategies (e.g., anhydrous caffeine capsules) may be preferable to multi-ingredient energy drinks when the aim is to isolate caffeine effects and improve dose standardization. Finally, consideration of female-specific factors (e.g., menstrual cycle and hormonal status) may help inform individualized supplementation decisions, but future research is required before these factors can be translated into definitive supplementation recommendations for female volleyball players.

## Data Availability

This study is based on data from previously published studies. The datasets generated through data extraction and analysis are included in the article and its Supplementary material. Further inquiries can be directed to the corresponding author.

## Footnotes

1
https://automeris.io/WebPlotDigitizer

## References

[1] Hulteen RM Smith JJ Morgan PJ Barnett LM Hallal PC Colyvas K . Global participation in sport and leisure-time physical activities: a systematic review and meta-analysis. Prev Med. (2017) 95:14–25. doi: 10.1016/j.ypmed.2016.11.027 27939265

[2] García-de-Alcaraz A Ramírez-Campillo R Rivera-Rodríguez M Romero-Moraleda B. Analysis of jump load during a volleyball season in terms of player role. J Sci Med Sport. (2020) 23:973–8. doi: 10.1016/j.jsams.2020.03.002, 32303475

[3] Sheppard J Newton R McGuigan M. The effect of accentuated eccentric load on jump kinetics in high-performance volleyball players. Int J Sports Sci Coach. (2007) 2:267–73. doi: 10.1260/174795407782233209

[4] Sheppard JM Cronin JB Gabbett TJ McGuigan MR Etxebarria N Newton RU. Relative importance of strength, power, and anthropometric measures to jump performance of elite volleyball players. J Strength Cond Res. (2008) 22:758–65. doi: 10.1519/JSC.0b013e31816a8440, 18438243

[5] Sattler T Sekulic D Hadzic V Uljevic O Dervisevic E. Vertical jumping tests in volleyball: reliability, validity, and playing-position specifics. J Strength Cond Res. (2012) 26:1532–8. doi: 10.1519/JSC.0b013e318234e838 21904238

[6] Forthomme B Croisier JL Ciccarone G Crielaard JM Cloes M. Factors correlated with volleyball spike velocity. Am J Sports Med. (2005) 33:1513–9. doi: 10.1177/0363546505274935, 16009986

[7] Alfredson H Pietilä T Lorentzon R. Concentric and eccentric shoulder and elbow muscle strength in female volleyball players and non-active females. Scand J Med Sci Sports. (1998) 8:265–70. doi: 10.1111/j.1600-0838.1998.tb00481.x, 9809384

[8] Tillman MD Hass CJ Brunt D Bennett GR. Jumping and landing techniques in elite women’s volleyball. J Sports Sci Med. (2004) 3:30. 24497818 PMC3896111

[9] Nagano Y Sasaki S Kose Y Ichikawa H. Detection of high-impact movements in a volleyball match: a cross-sectional study. Exercise Medicine. (2020) 4:3. doi: 10.26644/em.2020.003

[10] Baugh CM Weintraub GS Gregory AJ Djoko A Dompier TP Kerr ZY. Descriptive epidemiology of injuries sustained in national collegiate athletic association men’s and women’s volleyball, 2013-2014 to 2014-2015. Sports health. (2018) 10:60–9. doi: 10.1177/1941738117733685, 28985702 PMC5753967

[11] Tsarbou C Liveris NI Tsimeas PD Papageorgiou G Xergia SA Tsiokanos A. The effect of fatigue on jump height and the risk of knee injury after a volleyball training game: a pilot study. Biomed Hum Kinet. (2021) 13:197–204. doi: 10.2478/bhk-2021-0024

[12] Kim H Son S Seeley M Hopkins J. Functional fatigue alters lower-extremity neuromechanics during a forward-side jump. Int J Sports Med. (2015) 36:1192–200. doi: 10.1055/s-0035-1550050, 26422053

[13] O’Connor KM Johnson C Benson LC. The effect of isolated hamstrings fatigue on landing and cutting mechanics. J Appl Biomech. (2015) 31:211–20. doi: 10.1123/jab.2014-0098, 25781073

[14] Borotikar BS Newcomer R Koppes R McLean SG. Combined effects of fatigue and decision making on female lower limb landing postures: central and peripheral contributions to ACL injury risk. Clin Biomech. (2008) 23:81–92. doi: 10.1016/j.clinbiomech.2007.08.008, 17889972

[15] Hosseini SH Gheitasi M Hosseini SM. Effect of lower-limb fatigue on kinematic variables during an unanticipated block among adolescent female volleyball players. BMC Sports Sci Med Rehabil. (2025) 17:349. doi: 10.1186/s13102-025-01331-y, 41287021 PMC12642213

[16] Hernández-Landa RE Lazo M Salado DD Sánchez-Almanzar E Cepeda-Marte JL Zare R . Dietary supplementation strategies for improving training adaptations, antioxidant status and performance of volleyball players: a systematic review. J Sci Sport Exerc. (2024):1–21. doi: 10.1007/s42978-024-00297-6

[17] Slater GJ Sygo J Sprinting JM. Dietary approaches to optimize training adaptation and performance. Int J Sport Nutr Exerc Metab. (2019) 29:85–94. doi: 10.1123/ijsnem.2018-0273, 30943814

[18] Zhang Z Yin M Qiu B Meng F Wu B Wang Y . A systematic review and meta-analysis of the evidence on the acute effects of caffeine on sport-specific skills, physical performance, and physiological function in female basketball players. Front Nutr. (2026) 13:1766993. doi: 10.3389/fnut.2026.1766993, 41809105 PMC12968231

[19] Salinero JJ Lara B Del Coso J. Effects of acute ingestion of caffeine on team sports performance: a systematic review and meta-analysis. Res Sports Med. (2019) 27:238–56. doi: 10.1080/15438627.2018.1552146, 30518253

[20] Grgic J Grgic I Pickering C Schoenfeld BJ Bishop DJ Pedisic Z. Wake up and smell the coffee: caffeine supplementation and exercise performance-an umbrella review of 21 published meta-analyses. Br J Sports Med. (2020) 54:681–8. doi: 10.1136/bjsports-2018-100278, 30926628

[21] Kerksick CM Wilborn CD Roberts MD Smith-Ryan A Kleiner SM Jäger R . ISSN exercise & sports nutrition review update: research & recommendations. J Int Soc Sports Nutr. (2018) 15:38. doi: 10.1186/s12970-018-0242-y, 30068354 PMC6090881

[22] Southward K Rutherfurd-Markwick KJ Ali A. The effect of acute caffeine ingestion on endurance performance: a systematic review and meta-analysis. Sports Med. (2018) 48:1913–28. doi: 10.1007/s40279-018-0939-8, 29876876

[23] Del Coso J Muñoz G Muñoz-Guerra J. Prevalence of caffeine use in elite athletes following its removal from the world anti-doping agency list of banned substances. Appl Physiol Nutr Metab. (2011) 36:555–61. doi: 10.1139/h11-052, 21854160

[24] Weber A Herz R. The relationship between caffeine contracture of intact muscle and the effect of caffeine on reticulum. J Gen Physiol. (1968) 52:750–9. doi: 10.1085/jgp.52.5.750, 5688082 PMC2225844

[25] Reggiani C. Caffeine as a tool to investigate sarcoplasmic reticulum and intracellular calcium dynamics in human skeletal muscles. J Muscle Res Cell Motil. (2021) 42:281–9. doi: 10.1007/s10974-020-09574-7, 32034582

[26] Bloms LP Fitzgerald JS Short MW Whitehead JR. The effects of caffeine on vertical jump height and execution in collegiate athletes. J Strength Cond Res. (2016) 30:1855–61. doi: 10.1519/JSC.0000000000001280, 26626028

[27] Behrens M Mau-Moeller A Weippert M Fuhrmann J Wegner K Skripitz R . Caffeine-induced increase in voluntary activation and strength of the quadriceps muscle during isometric, concentric and eccentric contractions. Sci Rep. (2015) 5:10209. doi: 10.1038/srep10209, 25969895 PMC4429543

[28] Chen HY Chen YC Tung K Chao HH Wang HS. Effects of caffeine and sex on muscle performance and delayed-onset muscle soreness after exercise-induced muscle damage: a double-blind randomized trial. J Appl Physiol. 127:798–805. doi: 10.1152/japplphysiol.01108.2018 31219772

[29] Doherty M Smith PM. Effects of caffeine ingestion on rating of perceived exertion during and after exercise: a meta-analysis. Scand J Med Sci Sports. (2005) 15:69–78. doi: 10.1111/j.1600-0838.2005.00445.x, 15773860

[30] Doherty M Smith PM. Effects of caffeine ingestion on exercise testing: a meta-analysis. Int J Sport Nutr Exerc Metab. (2004) 14:626–46. doi: 10.1123/ijsnem.14.6.626, 15657469

[31] Kaszuba M Klocek O Spieszny M Filip-Stachnik A. The effect of caffeinated chewing gum on volleyball-specific skills and physical performance in volleyball players. Nutrients. (2022) 15:91. doi: 10.3390/nu15010091, 36615750 PMC9823551

[32] Fernández-Campos C Dengo AL Moncada-Jiménez J. Acute consumption of an energy drink does not improve physical performance of female volleyball players. Int J Sport Nutr Exerc Metab. (2015) 25:271–7. doi: 10.1123/ijsnem.2014-0101, 25387127

[33] Del Coso J Pérez-López A Abian-Vicen J Salinero JJ Lara B Valadés D. Enhancing physical performance in male volleyball players with a caffeine-containing energy drink. Int J Sports Physiol Perform. (2014) 9:1013–8. doi: 10.1123/ijspp.2013-0448, 24664858

[34] Zbinden-Foncea H Rada I Gomez J Kokaly M Stellingwerff T Deldicque L . Effects of caffeine on countermovement-jump performance variables in elite male volleyball players. Int J Sports Physiol Perform. (2018) 13:145–50. doi: 10.1123/ijspp.2016-0705, 28488924

[35] Nemati J Hemmatinafar M Niknam A Nikahd M Zeighami N Imanian B . Effects of different doses of caffeine supplementation on collegiate male volleyball players’ specific performance and skills: a randomized, double-blind, placebo-controlled, crossover study. Nutrients. (2023) 15:4049. doi: 10.3390/nu15184049, 37764832 PMC10536286

[36] Filip-Stachnik A Kaszuba M Dorozynski B Komarek Z Gawel D Del Coso J . Acute effects of caffeinated chewing gum on volleyball performance in high-performance female players. J Hum Kinet. (2022) 84:92–102. doi: 10.2478/hukin-2022-0092, 36457461 PMC9679174

[37] Filip-Stachnik A Spieszny M Stanisz L Krzysztofik M. Does caffeine ingestion affect the lower-body post-activation performance enhancement in female volleyball players? BMC Sports Sci Med Rehabil. (2022) 14:93. doi: 10.1186/s13102-022-00488-0, 35614511 PMC9131637

[38] dos Santos Vaz M. Caffeine improves volleyball serves precision among college male players. RPCD. (2016) 15:76–88. doi: 10.5628/rpcd.15.03.76

[39] Siquier-Coll J Delgado-García G Soto-Méndez F Liñán-González A García R González-Fernández FT. The effect of caffeine supplementation on female volleyball players’ performance and wellness during a regular training week. Nutrients. (2024) 16:29. doi: 10.3390/nu16010029, 38201859 PMC10780397

[40] Pérez-López A Salinero JJ Abian-Vicen J Valadés D Lara B Hernandez C . Caffeinated energy drinks improve volleyball performance in elite female players. Med Sci Sports Exerc. (2015) 47:850–6. doi: 10.1249/MSS.0000000000000455, 25051390

[41] Chen B Zhang C Xu Z Li Y Guo L Cao Y . Effects of caffeine supplementation on exercise performance in volleyball players: a systematic review and meta-analysis. Nutrients. (2025) 17:1709. doi: 10.3390/nu17101709, 40431449 PMC12113779

[42] Negaresh R Al-Riyami SAA Paahoo A Hoseini R Coso JD. Effects of acute caffeine ingestion on physical performance and skill execution in volleyball players: a systematic review and meta-analysis. Int J Exerc Sci. (2025) 18:922–48. doi: 10.70252/FRCN1471, 41079003 PMC12510704

[43] Julian R Hecksteden A Fullagar HHK Meyer T. The effects of menstrual cycle phase on physical performance in female soccer players. PLoS One. (2017) 12:e0173951. doi: 10.1371/journal.pone.0173951, 28288203 PMC5348024

[44] Tounsi M Jaafar H Aloui A Souissi N. Soccer-related performance in eumenorrheic tunisian high-level soccer players: effects of menstrual cycle phase and moment of day. J Sports Med Phys Fitness. (2018) 58:497–502. doi: 10.23736/S0022-4707.17.06958-4, 28222573

[45] Ribeiro-Alves MA Trugo LC Donangelo CM. Use of oral contraceptives blunts the calciuric effect of caffeine in young adult women. J Nutr. (2003) 133:393–8. doi: 10.1093/jn/133.2.393, 12566473

[46] Goldstein ER. The Effects of Caffeine Supplementation on Strength And Muscular Endurance in Resistance-Trained Females. United States -- Florida: Florida Atlantic University (2009).

[47] Mielgo-Ayuso J Marques-Jiménez D Refoyo I Del Coso J León-Guereño P Calleja-González J. Effect of caffeine supplementation on sports performance based on differences between sexes: a systematic review. Nutrients. (2019) 11:2313–3. doi: 10.3390/nu11102313, 31574901 PMC6835847

[48] Gomez-Bruton A Marin-Puyalto J Muñiz-Pardos B Matute-Llorente A Del Coso J Gomez-Cabello A . Does acute caffeine supplementation improve physical performance in female team-sport athletes? Evidence from a systematic review and meta-analysis. Nutrients. (2021) 13:3663. doi: 10.3390/nu13103663, 34684665 PMC8538965

[49] Page MJ McKenzie JE Bossuyt PM Boutron I Hoffmann TC Mulrow CD . The PRISMA 2020 statement: an updated guideline for reporting systematic reviews. BMJ. 372:n71. doi: 10.1136/bmj.n71, 33782057 PMC8005924

[50] Lee CL Cheng CF Astorino TA Lee CJ Huang HW Chang WD. Effects of carbohydrate combined with caffeine on repeated sprint cycling and agility performance in female athletes. J Int Soc Sports Nutr. (2014) 11:17. doi: 10.1186/1550-2783-11-17, 24855458 PMC4012529

[51] Cumpston M Li T Page MJ Chandler J Welch VA Higgins JP . Updated guidance for trusted systematic reviews: a new edition of the Cochrane handbook for systematic reviews of interventions. Cochrane Database Syst Rev. (2019) 10:ED000142. doi: 10.1002/14651858.ED000142, 31643080 PMC10284251

[52] Sterne JA Savović J Page MJ Elbers RG Blencowe NS Boutron I . RoB 2: a revised tool for assessing risk of bias in randomised trials. BMJ. (2019) 366:l4898. doi: 10.1136/bmj.l4898, 31462531

[53] de Morton NA. The PEDro scale is a valid measure of the methodological quality of clinical trials: a demographic study. Aust J Physiother. (2009) 55:129–33. doi: 10.1016/s0004-9514(09)70043-1, 19463084

[54] Guyatt GH Oxman AD Vist GE Kunz R Falck-Ytter Y Alonso-Coello P . GRADE: an emerging consensus on rating quality of evidence and strength of recommendations. BMJ. (2008) 336:924–6. doi: 10.1136/bmj.39489.470347.AD, 18436948 PMC2335261

[55] DerSimonian R Laird N. Meta-analysis in clinical trials. Control Clin Trials. (1986) 7:177–88. doi: 10.1016/0197-2456(86)90046-2, 3802833

[56] Van den Noortgate W López-López JA Marín-Martínez F Sánchez-Meca J. Three-level meta-analysis of dependent effect sizes. Behav Res Methods. (2013) 45:576–94. doi: 10.3758/s13428-012-0261-6, 23055166

[57] Konstantopoulos S. Fixed effects and variance components estimation in three-level meta-analysis. Res Synth Methods. (2011) 2:61–76. doi: 10.1002/jrsm.35, 26061600

[58] Lakens D. Calculating and reporting effect sizes to facilitate cumulative science: a practical primer for t-tests and ANOVAs. Front Psychol. (2013) 4:863. doi: 10.3389/fpsyg.2013.00863, 24324449 PMC3840331

[59] Nagashima K Noma H Furukawa TA. Prediction intervals for random-effects meta-analysis: a confidence distribution approach. Stat Methods Med Res. (2019) 28:1689–702. doi: 10.1177/0962280218773520, 29745296

[60] Higgins JPT Thompson SG Deeks JJ Altman DG. Measuring inconsistency in meta-analyses. BMJ. (2003) 327:557–60. doi: 10.1136/bmj.327.7414.557, 12958120 PMC192859

[61] Borenstein M Hedges LV Higgins JP Rothstein HR. Introduction to Meta-Analysis. Hoboken, New Jersey, USA: John Wiley & Sons (2021).

[62] Aung NM Jurak I Mehmood S Axon E. Sensitivity analysis in meta-analysis: a tutorial. Cochrane Evid Synth Methods. (2026) 4:e70067. doi: 10.1002/cesm.70067, 41497796 PMC12767030

[63] McGuinness LA Higgins JPT. Risk-of-bias VISualization (robvis): an R package and shiny web app for visualizing risk-of-bias assessments. Res Synth Methods. (2021) 12:55–61. doi: 10.1002/jrsm.1411, 32336025

[64] Peña J Rodríguez-Guerra J Serra N. Which skills and factors better predict winning and losing in high-level men’s volleyball? J Strength Cond Res. (2013) 27:2487–93. doi: 10.1519/JSC.0b013e31827f4dbe, 23222090

[65] Diedhiou AB Erkan D Guler M Sar H Karakulak I Eyuboglu E . The effect of low dose caffeine powder supplementation on serve speed, spike speed, and speed-endurance in elite sitting volleyball players: a randomized double-blind crossover study. BMC Sports Sci Med Rehabil. (2025) 17:320. doi: 10.1186/s13102-025-01408-8, 41199325 PMC12593936

[66] Oliveira L d S Moura TBMA Rodacki ALF Tilp M Okazaki VHA. A systematic review of volleyball spike kinematics: implications for practice and research. Int J Sports Sci Coach. (2020) 15:239–55. doi: 10.1177/1747954119899881

[67] Slovák L Sarvestan J Iwatsuki T Zahradník D Land WM Abdollahipour R. External focus of attention enhances arm velocities during volleyball spike in young female players. Front Psychol. (2023) 13:1041871. doi: 10.3389/fpsyg.2022.1041871, 36687905 PMC9851077

[68] Karayigit R Koz M Sánchez-Gómez A Naderi A Yildirim UC Domínguez R . High dose of caffeine mouth rinse increases resistance training performance in men. Nutrients. (2021) 13:3800. doi: 10.3390/nu13113800, 34836058 PMC8617760

[69] Guest NS VanDusseldorp TA Nelson MT Grgic J Schoenfeld BJ Jenkins NDM . International society of sports nutrition position stand: caffeine and exercise performance. J Int Soc Sports Nutr. (2021) 18:1. doi: 10.1186/s12970-020-00383-4, 33388079 PMC7777221

[70] Fredholm BB Bättig K Holmén J Nehlig A Zvartau EE. Actions of caffeine in the brain with special reference to factors that contribute to its widespread use. Pharmacol Rev. (1999) 51:83–133. doi: 10.1016/S0031-6997(24)01396-6, 10049999

[71] Finocchi C Ferrari M. Female reproductive steroids and neuronal excitability. Neurol Sci. (2011) 32:31–5. doi: 10.1007/s10072-011-0532-5 21533709

[72] Grgic J Varovic D. Moderators of caffeine’s effects on jumping performance in females: a systematic review and Meta-analysis. J Am Nutr Assoc. (2024) 43:92–100. doi: 10.1080/27697061.2023.2212740, 37191618

[73] Lindinger M Willmets R Hawke T. Stimulation of na+, K+-pump activity in skeletal muscle by methylxanthines: evidence and proposed mechanisms. Acta Physiol Scand. (1996) 156:347–53. doi: 10.1046/j.1365-201X.1996.200000.x, 8729695

[74] Granfors MT Backman JT Laitila J Neuvonen PJ. Oral contraceptives containing ethinyl estradiol and gestodene markedly increase plasma concentrations and effects of tizanidine by inhibiting cytochrome P450 1A2. Clin. Pharmacol. Ther. (2005) 78:400–11. doi: 10.1016/j.clpt.2005.06.009, 16198659

[75] Lundsgaard AM Kiens B. Gender differences in skeletal muscle substrate metabolism – molecular mechanisms and insulin sensitivity. Front Endocrinol. (2014) 5:195. doi: 10.3389/fendo.2014.00195, 25431568 PMC4230199

[76] James JJ Mellow ML Bueckers EP Gutsch SB Wrucke DJ Pearson AG . Sex differences in human skeletal muscle fiber types and the influence of age, physical activity, and muscle group: a systematic review and meta-analysis. Physiol Rep. (2025) 13:e70616. doi: 10.14814/phy2.70616, 41178056 PMC12580412

[77] Ja D. The 505 test: a test for agility in horizontal plane. Aust J Sci Med Sport. (1985) 17:15–8.

[78] Semenick D. Tests and measurements: the T-test. Natl Strength Cond Assoc J. (1990) 12:36–7. doi: 10.1519/0744-0049(1990)012<0036:TTT>2.3.CO;2

[79] Wang J Dewi L Peng Y Hou CW Song Y Condello G. Does ergogenic effect of caffeine supplementation depend on CYP1A2 genotypes? A systematic review with meta-analysis. J Sport Health Sci. (2024) 13:499–508. doi: 10.1016/j.jshs.2023.12.005, 38158179 PMC11184386

[80] Filip-Stachnik A Krzysztofik M Del Coso J Wilk M. Acute effects of high doses of caffeine on bar velocity during the bench press throw in athletes habituated to caffeine: a randomized, double-blind and crossover study. J Clin Med. (2021) 10:4380. doi: 10.3390/jcm10194380, 34640398 PMC8509759

[81] Turnbull D Rodricks JV Mariano GF. Neurobehavioral hazard identification and characterization for caffeine. Regul Toxicol Pharmacol. (2016) 74:81–92. doi: 10.1016/j.yrtph.2015.12.002, 26702789

[82] Bendlin BB Trouard TP Ryan L. Caffeine attenuates practice effects in word stem completion as measured by fMRI BOLD signal. Hum Brain Mapp. (2007) 28:654–62. doi: 10.1002/hbm.20295, 17094121 PMC6871275

[83] Lazić A Kocić M Trajković N Popa C Peyré-Tartaruga LA Padulo J. Acute effects of caffeine on overall performance in basketball players—a systematic review. Nutrients. (2022) 14:1930–17. doi: 10.3390/nu14091930, 35565897 PMC9099691

[84] Abian-Vicen J Puente C Salinero JJ González-Millán C Areces F Muñoz G . A caffeinated energy drink improves jump performance in adolescent basketball players. Amino Acids. (2014) 46:1333–41. doi: 10.1007/s00726-014-1702-6, 24599611

[85] Del Coso J Muñoz-Fernández VE Muñoz G Fernández-Elías VE Ortega JF Hamouti N . Effects of a caffeine-containing energy drink on simulated soccer performance. PLoS One. (2012) 7:e31380. doi: 10.1371/journal.pone.0031380, 22348079 PMC3279366

[86] Del Coso J Portillo J Muñoz G Abián-Vicén J Gonzalez-Millán C Muñoz-Guerra J. Caffeine-containing energy drink improves sprint performance during an international rugby sevens competition. Amino Acids. (2013) 44:1511–9. doi: 10.1007/s00726-013-1473-5, 23462927

[87] Zhi Sen T Sim A Kawabata M Burns SF. A systematic review of the effects of caffeine on basketball performance outcomes. Biology (Basel). (2022) 11:17. doi: 10.3390/biology11010017, 35053015 PMC8773249

[88] Gliottoni RC Motl RW. Effect of caffeine on leg-muscle pain during intense cycling exercise: possible role of anxiety sensitivity. Int J Sport Nutr Exerc Metab. (2008) 18:103–15. doi: 10.1123/ijsnem.18.2.103, 18458355

[89] Duncan MJ Stanley M Parkhouse N Cook K Smith M. Acute caffeine ingestion enhances strength performance and reduces perceived exertion and muscle pain perception during resistance exercise. Eur J Sport Sci. (2013) 13:392–9. doi: 10.1080/17461391.2011.635811, 23834545

[90] Karayigit R Forbes SC Osmanov Z Yilmaz C Yasli BC Naderi A . Low and moderate doses of caffeinated coffee improve repeated sprint performance in female team sport athletes. Biology (Basel). (2022) 11:1498. doi: 10.3390/biology11101498, 36290401 PMC9598515

[91] Arnold MA Carr DB Togasaki DM Pian MC Martin JB. Caffeine stimulates β-endorphin release in blood but not in cerebrospinal fluid. Life Sci. (1982) 31:1017–24. doi: 10.1016/0024-3205(82)90174-6, 6290811

[92] Roelands B Buyse L Pauwels F Delbeke F Deventer K Meeusen R. No effect of caffeine on exercise performance in high ambient temperature. Eur J Appl Physiol. (2011) 111:3089–95. doi: 10.1007/s00421-011-1945-9, 21461761

[93] Bakan K Ayca IB Oral O. The effect of caffeine use in elite female basketball players on blood pressure, heart rate and accurate shot. Sci Chronicles. (2024) 29:460–70.

[94] Raya-González J Scanlan AT Soto-Célix M Rodríguez-Fernández A Castillo D. Caffeine ingestion improves performance during fitness tests but does not alter activity during simulated games in professional basketball players. Int J Sports Physiol Perform. (2021) 16:387–94. doi: 10.1123/ijspp.2020-0144, 33401238

[95] Puente C Abián-Vicén J Salinero JJ Lara B Areces F Del Coso J. Caffeine improves basketball performance in experienced basketball players. Nutrients. (2017) 9:1033. doi: 10.3390/nu9091033, 28925969 PMC5622793

[96] Douligeris A Methenitis S Lazou A Panayiotou G Feidantsis K Voulgaridou G . The effect of acute pre-workout supplement ingestion on basketball-specific performance of well-trained athletes. Nutrients. (2023) 15:2304. doi: 10.3390/nu15102304, 37242187 PMC10220844

[97] Diler K Örer GE. The effect of caffeine consumed before competition on heart rate trigger squeeze time and shooting score in air pistol athletes. Pak J Med Health Sci. (2021) 15:3202–5. doi: 10.53350/pjmhs2115113202

[98] Tan ZS Burns SF Pan JW Kong PW. Effect of caffeine ingestion on free-throw performance in college basketball players. J Exerc Sci Fit. (2020) 18:62–7. doi: 10.1016/j.jesf.2019.12.002, 31908649 PMC6939085

[99] Nana A Ramyarangsi P Jamwai L Hiranphan P Siripornpanich V Ajjimaporn A. Low-dose caffeine enhances cognitive processing but not physical performance in fatigued taekwondo athletes: a randomized crossover trial. J Int Soc Sports Nutr. (2025) 22:2526094. doi: 10.1080/15502783.2025.2526094, 40581775 PMC12207766

[100] Bezuglov E Vakhidov T Malyakin G Kapralova E Emanov A Koroleva E . The influence of caffeine on tolerance to sport-specific high-intensity exercise in young elite soccer players. J Hum Nutr Diet. (2025) 38:1–11. doi: 10.1111/jhn.70002, 39718415

[101] Kazan HH Bulgay C Zorba E Dalip M Ceylan HI Semenova EA . Exploring the relationship between caffeine metabolism-related CYP1A2 rs762551 polymorphism and team sport athlete status and training adaptations. Mol Biol Rep. (2024) 51:841. doi: 10.1007/s11033-024-09800-2, 39042267 PMC11266271

[102] Chia JS Barrett LA Chow JY Burns SF. Effects of caffeine supplementation on performance in ball games. Sports Med. (2017) 47:2453–71. doi: 10.1007/s40279-017-0763-6, 28741186

[103] Magkos F Kavouras SA. Caffeine use in sports, pharmacokinetics in man, and cellular mechanisms of action. Crit Rev Food Sci Nutr. (2005) 45:535–62. doi: 10.1080/1040-830491379245, 16371327

[104] Morris C Viriot SM Farooq Mirza QUA Morris GA Lynn A. Caffeine release and absorption from caffeinated gums. Food Funct. (2019) 10:1792–6. doi: 10.1039/c9fo00431a, 30919868

[105] Filip-Stachnik A. Acute effects of caffeinated chewing gum on basketball performance in elite female players. J Kinesiol Exerc Sci. (2022) 32:22–30. doi: 10.5604/01.3001.0016.1233

[106] Nakamura Y Aizawa K. "Sex hormones, menstrual cycle, and resistance exercise". In: Sex Hormones, Exercise and Women: Scientific and Clinical Aspects Cham, Switzerland: Springer (2023). p. 227–43.

[107] Enns DL Tiidus PM. The influence of estrogen on skeletal muscle: sex matters. Sports Med. (2010) 40:41–58. doi: 10.2165/11319760-000000000-00000, 20020786

[108] Grgic J. Exploring the minimum ergogenic dose of caffeine on resistance exercise performance: a meta-analytic approach. Nutrition. (2022) 97:111604. doi: 10.1016/j.nut.2022.111604, 35203046

[109] Grgic J. Effect of low caffeine doses on jumping performance: a meta-analysis. Nutr Food Sci. (2022) 53:50–60. doi: 10.1108/NFS-02-2022-0050, 35579975

