# Supplementary material for: Effects of acute caffeine supplementation on physical, physiological, and sport-specific performance in female volleyball players: a systematic review and three-level meta-analysis
Source: Front Nutr. 2026 Jul 8;13:1880118. doi: 10.3389/fnut.2026.1880118 (PMC13388447; doi:10.3389/fnut.2026.1880118)
Supplement: Supplementary file 1 [file Supplementary_file_1.docx]

Supplementary Material

# Supplementary Table 1. PRISMA 2020 Checklist

| **Section and Topic** | **Item #** | **Checklist item** | **Location where item is reported** |
| --- | --- | --- | --- |
| **TITLE** | | |  |
| Title | 1 | Identify the report as a systematic review. | Title |
| **ABSTRACT** | | |  |
| Abstract | 2 | See the PRISMA 2020 for Abstracts checklist. | Abstract |
| **INTRODUCTION** | | |  |
| Rationale | 3 | Describe the rationale for the review in the context of existing knowledge. | Background |
| Objectives | 4 | Provide an explicit statement of the objective(s) or question(s) the review addresses. | Objectives |
| **METHODS** | | |  |
| Eligibility criteria | 5 | Specify the inclusion and exclusion criteria for the review and how studies were grouped for the syntheses. | Inclusion and exclusion criteria |
| Information sources | 6 | Specify all databases, registers, websites, organisations, reference lists and other sources searched or consulted to identify studies. Specify the date when each source was last searched or consulted. | Search strategy |
| Search strategy | 7 | Present the full search strategies for all databases, registers and websites, including any filters and limits used. | Search strategy Figure 1 |
| Selection process | 8 | Specify the methods used to decide whether a study met the inclusion criteria of the review, including how many reviewers screened each record and each report retrieved, whether they worked independently, and if applicable, details of automation tools used in the process. | Search strategy Figure 1 |
| Data collection process | 9 | Specify the methods used to collect data from reports, including how many reviewers collected data from each report, whether they worked independently, any processes for obtaining or confirming data from study investigators, and if applicable, details of automation tools used in the process. | Data extraction and transformation |
| Data items | 10a | List and define all outcomes for which data were sought. Specify whether all results that were compatible with each outcome domain in each study were sought (e.g. for all measures, time points, analyses), and if not, the methods used to decide which results to collect. | Data extraction and transformation |
|  | 10b | List and define all other variables for which data were sought (e.g. participant and intervention characteristics, funding sources). Describe any assumptions made about any missing or unclear information. | Table 1 |
| Study risk of bias assessment | 11 | Specify the methods used to assess risk of bias in the included studies, including details of the tool(s) used, how many reviewers assessed each study and whether they worked independently, and if applicable, details of automation tools used in the process. | Quality assessment of included studies |
| Effect measures | 12 | Specify for each outcome the effect measure(s) (e.g. risk ratio, mean difference) used in the synthesis or presentation of results. | Statistical analysis |
| Synthesis methods | 13a | Describe the processes used to decide which studies were eligible for each synthesis (e.g. tabulating the study intervention characteristics and comparing against the planned groups for each synthesis (item #5)). | Statistical analysis |
|  | 13b | Describe any methods required to prepare the data for presentation or synthesis, such as handling of missing summary statistics, or data conversions. | Statistical analysis |
|  | 13c | Describe any methods used to tabulate or visually display results of individual studies and syntheses. | Statistical analysis |
|  | 13d | Describe any methods used to synthesize results and provide a rationale for the choice(s). If meta-analysis was performed, describe the model(s), method(s) to identify the presence and extent of statistical heterogeneity, and software package(s) used. | Statistical analysis |
|  | 13e | Describe any methods used to explore possible causes of heterogeneity among study results (e.g. subgroup analysis, meta-regression). | Statistical analysis |
|  | 13f | Describe any sensitivity analyses conducted to assess robustness of the synthesized results. | Statistical analysis |
| Reporting bias assessment | 14 | Describe any methods used to assess risk of bias due to missing results in a synthesis (arising from reporting biases). | N/A |
| Certainty assessment | 15 | Describe any methods used to assess certainty (or confidence) in the body of evidence for an outcome. | Statistical analysis |
| **RESULTS** | | |  |
| Study selection | 16a | Describe the results of the search and selection process, from the number of records identified in the search to the number of studies included in the review, ideally using a flow diagram. | Literature screening process; Figure 1 |
|  | 16b | Cite studies that might appear to meet the inclusion criteria, but which were excluded, and explain why they were excluded. | Literature screening process; Figure 1 |
| Study characteristics | 17 | Cite each included study and present its characteristics. | Participants and studies characteristics |
| Risk of bias in studies | 18 | Present assessments of risk of bias for each included study. | Risk of bias assessment in included studies; Figure 2 |
| Results of individual studies | 19 | For all outcomes, present, for each study: (a) summary statistics for each group (where appropriate) and (b) an effect estimate and its precision (e.g. confidence/credible interval), ideally using structured tables or plots. | Meta-analysis results Supplementary Figure 1 |
| Results of syntheses | 20a | For each synthesis, briefly summarise the characteristics and risk of bias among contributing studies. | Meta-analysis results |
|  | 20b | Present results of all statistical syntheses conducted. If meta-analysis was done, present for each the summary estimate and its precision (e.g. confidence/credible interval) and measures of statistical heterogeneity. If comparing groups, describe the direction of the effect. | Meta-analysis results |
|  | 20c | Present results of all investigations of possible causes of heterogeneity among study results. | Meta-analysis results |
|  | 20d | Present results of all sensitivity analyses conducted to assess the robustness of the synthesized results. | Supplementary Table 5 |
| Reporting biases | 21 | Present assessments of risk of bias due to missing results (arising from reporting biases) for each synthesis assessed. | N/A |
| Certainty of evidence | 22 | Present assessments of certainty (or confidence) in the body of evidence for each outcome assessed. | Supplementary Table 4 |
| **DISCUSSION** | | |  |
| Discussion | 23a | Provide a general interpretation of the results in the context of other evidence. | Evidence summary |
|  | 23b | Discuss any limitations of the evidence included in the review. | Study limitations |
|  | 23c | Discuss any limitations of the review processes used. | Study limitations |
|  | 23d | Discuss implications of the results for practice, policy, and future research. | Practical applications |
| **OTHER INFORMATION** | | |  |
| Registration and protocol | 24a | Provide registration information for the review, including register name and registration number, or state that the review was not registered. | Search strategy |
|  | 24b | Indicate where the review protocol can be accessed, or state that a protocol was not prepared. | Search strategy |
|  | 24c | Describe and explain any amendments to information provided at registration or in the protocol. | N/A |
| Support | 25 | Describe sources of financial or non-financial support for the review, and the role of the funders or sponsors in the review. | Funding |
| Competing interests | 26 | Declare any competing interests of review authors. | Conflict of interest |
| Availability of data, code and other materials | 27 | Report which of the following are publicly available and where they can be found: template data collection forms; data extracted from included studies; data used for all analyses; analytic code; any other materials used in the review. | Data availability statement |

# Supplementary Table 2. Physiotherapy Evidence Database (PEDro)

| **Study** | **The first author** | **Year of Publication** | **D1** | **D2** | **D3** | **D4** | **D5** | **D6** | **D7** | **D8** | **D9** | **D10** | **D11** | **Total** |  |
| --- | --- | --- | --- | --- | --- | --- | --- | --- | --- | --- | --- | --- | --- | --- | --- |
| 1 | Perez-López | 2015 | Y | 1 | 1 | 1 | 1 | 1 | 1 | 1 | 0 | 1 | 1 | 9 |  |
| 2 | Fernández | 2015 | Y | 1 | 1 | 1 | 1 | 0 | 0 | 1 | 0 | 1 | 1 | 7 |  |
| 3 | Filip Stachnik (a) | 2022 | Y | 1 | 1 | 1 | 1 | 0 | 0 | 1 | 1 | 1 | 1 | 8 |  |
| 4 | Filip Stachnik (b) | 2022 | Y | 1 | 1 | 1 | 1 | 1 | 0 | 1 | 0 | 1 | 1 | 9 |  |
| 5 | Siquier | 2023 | Y | 1 | 0 | 1 | 1 | 0 | 0 | 1 | 1 | 1 | 1 | 7 |  |
| **Table note:** “Y” or “1” indicates that the study meets the criterion; “N” or “0” indicates that the study does not meet the criterion. | | | | | | | | | | | | | | | |

# Supplementary Table 3. Risk bias assessment tool RoB2

| **Study** | **D1** | **D2** | **D3** | **D4** | **D5** | **Overall** |
| --- | --- | --- | --- | --- | --- | --- |
| Perez-López et al 2015 | Low | Low | Low | Low | Some concerns | Some concerns |
| Fernández et al 2015 | Low | Low | Some concerns | Low | Some concerns | Some concerns |
| Filip Stachnik et al (a) 2022 | Low | Low | Low | Low | Some concerns | Some concerns |
| Filip Stachnik et al (b) 2022 | Low | Low | Some concerns | Low | Some concerns | Some concerns |
| Siquier et al 2023 | Some concerns | Low | Low | Low | Some concerns | Some concerns |

# Supplementary Table 4. GRADE-based evidence rating for the conclusions of this study

| **Outcome** | **No of participants (Studies)** | **Certainty Assessment** | | | | | **Standardized Mean**  **effect (95% CI) †** | **GRADE*** |
| --- | --- | --- | --- | --- | --- | --- | --- | --- |
|  |  | **Risk of Bias** | **Inconsistency** | **Indirectness** | **Imprecision** | **Other** |  |  |
| **Caffeine (CAF) *versus* Placebo (PLA)** | | | | | | | |  |
| **Spiking Performance** | **150 (2 RCT)** | **Some serious** | **Not serious** | **Not serious** | **Serious** | **None** | **0.17（-0.43 to 0.75）** | **⨁◯◯◯**  **Very low** |
| **Jump height performance** | **490 (4 RCT)** | **Some serious** | **Not serious** | **Not serous** | **Some serious** | **None** | **0.16（−0.43 to 0.17）** | **⨁⨁◯◯ Low** |
| **Change-of-direction performance (Agility)** | **74 (2 RCT)** | **Some serious** | **Not serious** | **Not serious** | **Serious** | **None** | **0.26 ( -1.16 to 0.65 )** | **⨁◯◯◯**  **Very low** |
| **Handgrip strength performance** | **224 (3 RCT)** | **Some serious** | **Not serious** | **Not serious** | **Serious** | **None** | **0.29 ( −0.38 to 0.95 )** | **⨁◯◯◯ Very low** |
| **Power output** | **154 (2 RCT)** | **Some serious** | **Not serious** | **Not serious** | **Serious** | **None** | **0.16 ( −0.43 to 0.74 )** | **⨁◯◯◯ Very low** |
| **Fatigue-related outcomes** | **160 (3 RCT)** | **Some serious** | **Not serious** | **Not serious** | **Serious** | **None** | **0.09 ( −0.59 to 0.78)** | **⨁◯◯◯ Very low** |
| **Heart Rate** | **100 (2 RCT)** | **Some serious** | **Not serious** | **Not serious** | **Serious** | **None** | **0.33 ( −0.70 to 1.36)** | **⨁◯◯◯ Very low** |
| ***Certainty of evidence according to Grading of Recommendation, Assessment, Development, and Evaluations (GRADE):**  **Hight: We are very confidence in the estimated effect**  **Moderate: Our confidence in the estimated effect is moderate**  **Low: We have limited confidence in the estimate effect**  **Very low: We have very limited confidence in the estimate of effect**  **No of participants: Total number of participants with pooled effects** | | | | | | | | |

# Supplementary Table 5. Leave-One-Out Sensitivity Analysis: Influence of Individual Studies on Pooled Effect Estimates

| **Study Omitted** | **Number of Remaining Studies** | **SMD(95% CI)** | **p-value** | **I² (%)** | **Direction Consistent** | **Significance Change** |
| --- | --- | --- | --- | --- | --- | --- |
| **Jump height performance** | | | | | | |
| **None** | 4 | 0.16 (−0.43, 0.17) | 0.505 | 0 | N/A | N/A |
| **Pérez-López et al. 2015** | 3 | 0.26 (-0.16, 0.67) | 0.208 | 10.6 | No | No |
| **Fernández et al. 2015** | 3 | 0.17 (-0.27, 0.60) | 0.434 | 6.5 | No | No |
| **Filip‑Stachnik et al. 2022 (a)** | 3 | 0.37 (-0.02, 0.77) | 0.061 | 0 | No | No |
| **Siquier et al. 2023** | 3 | 0.26 (-0.14, 0.67) | 0.177 | 0 | No | No |
| **Handgrip strength performance** | | | | | | |
| **None** | 3 | 0.29 (−0.38, 0.95) | 0.352 | 44.4 | N/A | N/A |
| **Pérez-López et al. 2015** | 2 | 0.01 (-0.53, 0.55) | 0.970 | 0 | No | No |
| **Fernández et al. 2015** | 2 | 0.45 (-0.68, 1.57) | 0.379 | 58.1 | No | No |
| **Siquier et al. 2023** | 2 | 0.45 (-0.95, 1.84) | 0.383 | 67.9 | No | No |
| **Fatigue-related outcomes** | | | | | | |
| **None** | 3 | 0.09 (−0.59, 0.78) | 0.757 | 46.4 | N/A | N/A |
| **Pérez-López et al. 2015** | 2 | -0.09 (-0.89, 0.70) | 0.784 | 41.6 | **Yes** | No |
| **Fernández et al. 2015** | 2 | 0.29 (-0.55, 1.12) | 0.435 | 41,4 | No | No |
| **Siquier et al. 2023** | 2 | 0.11 (-6.31, 6.54) | 0.861 | 73.8 | No | No |

# Supplementary Figure 1. Forest plots for each outcome.

**
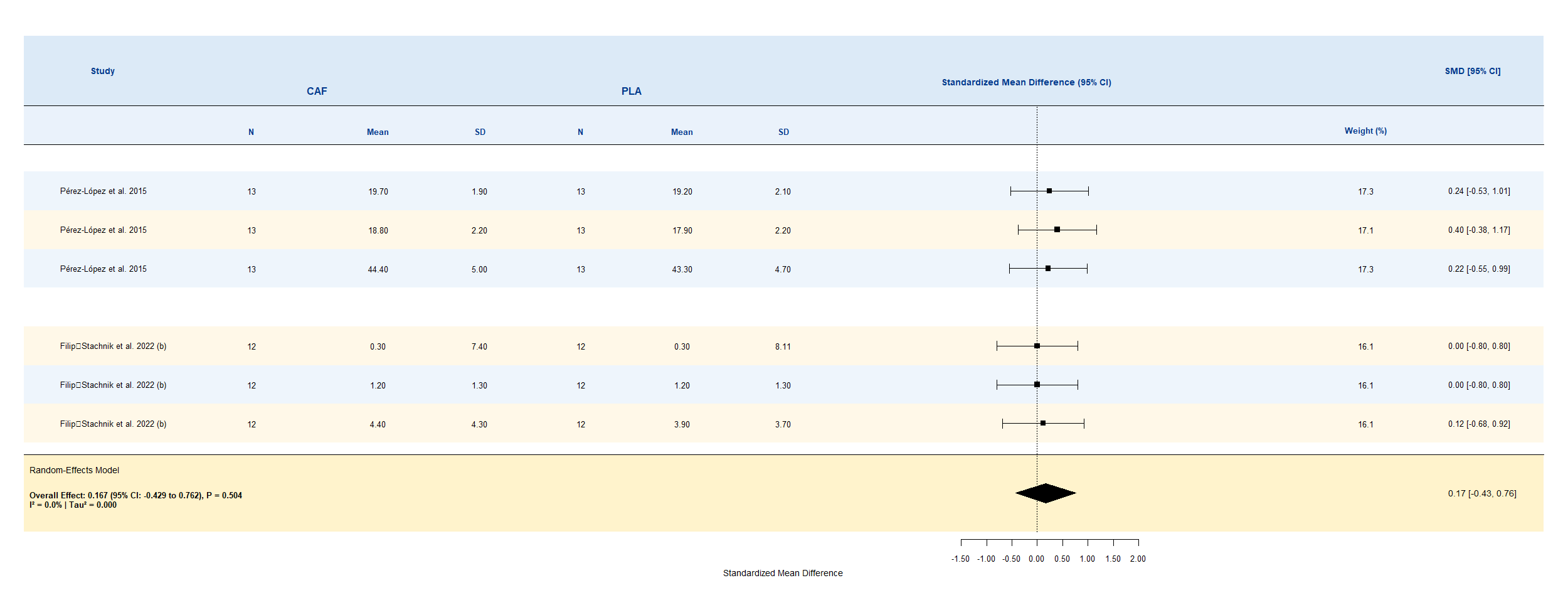
6.1 Spiking Performance**

**
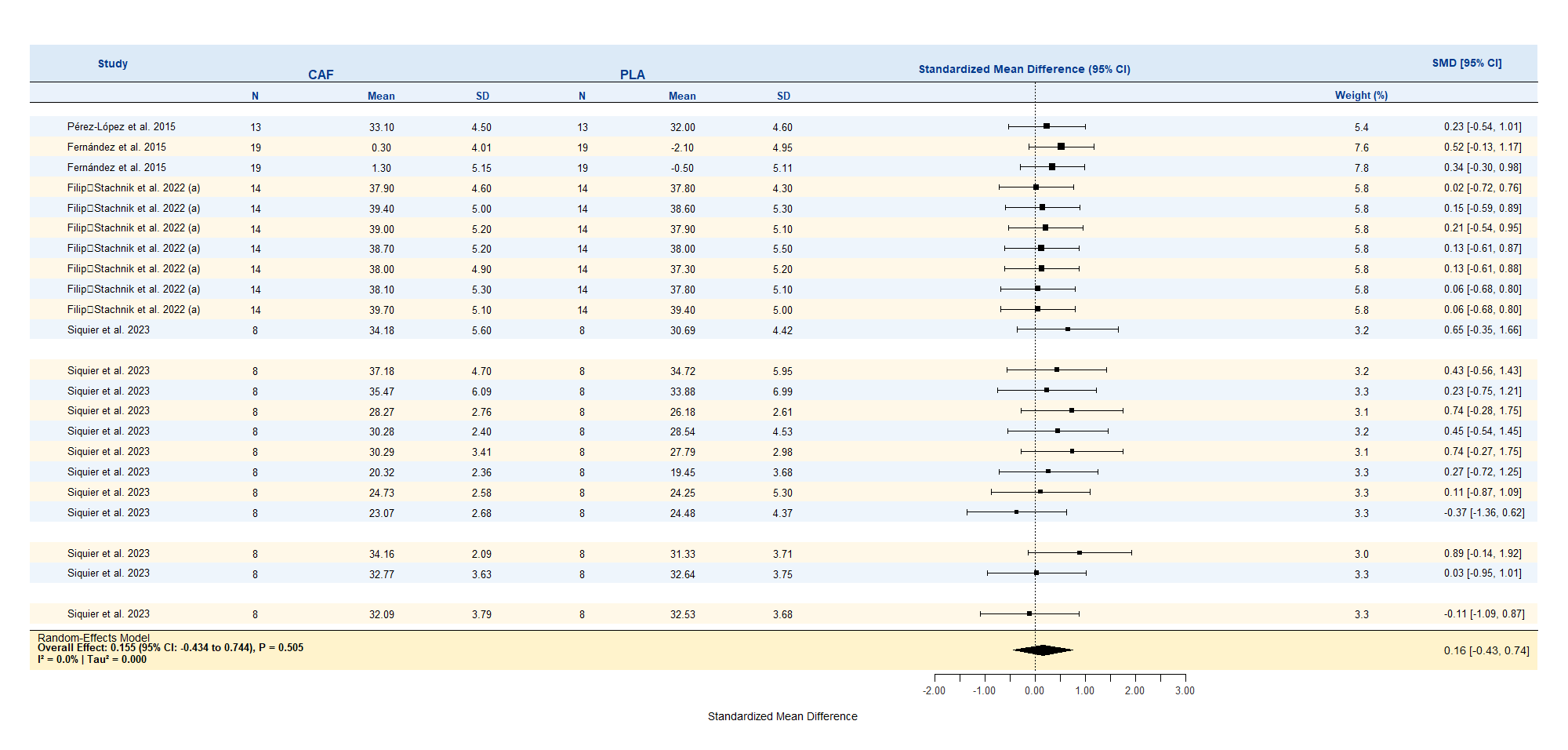
6.2 Jump height performance**

**
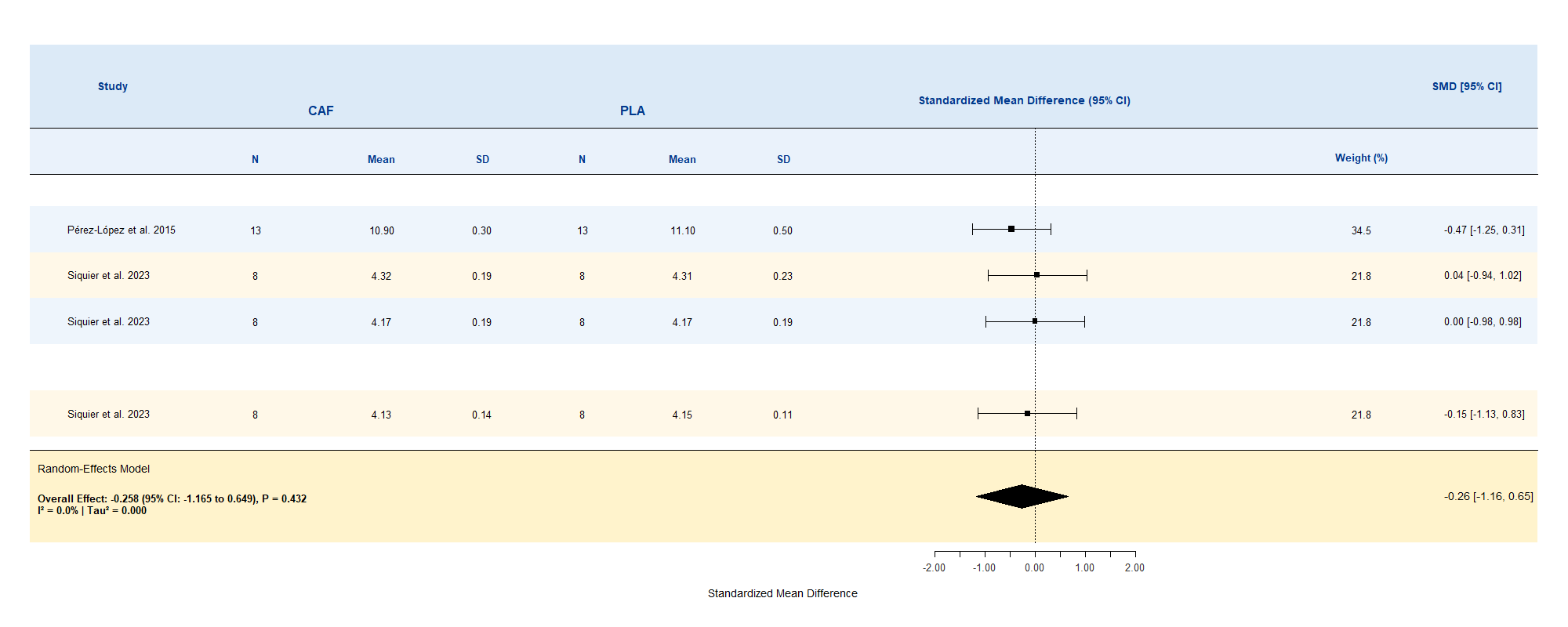
6.3 Change-of-direction performance (Agility)**

**
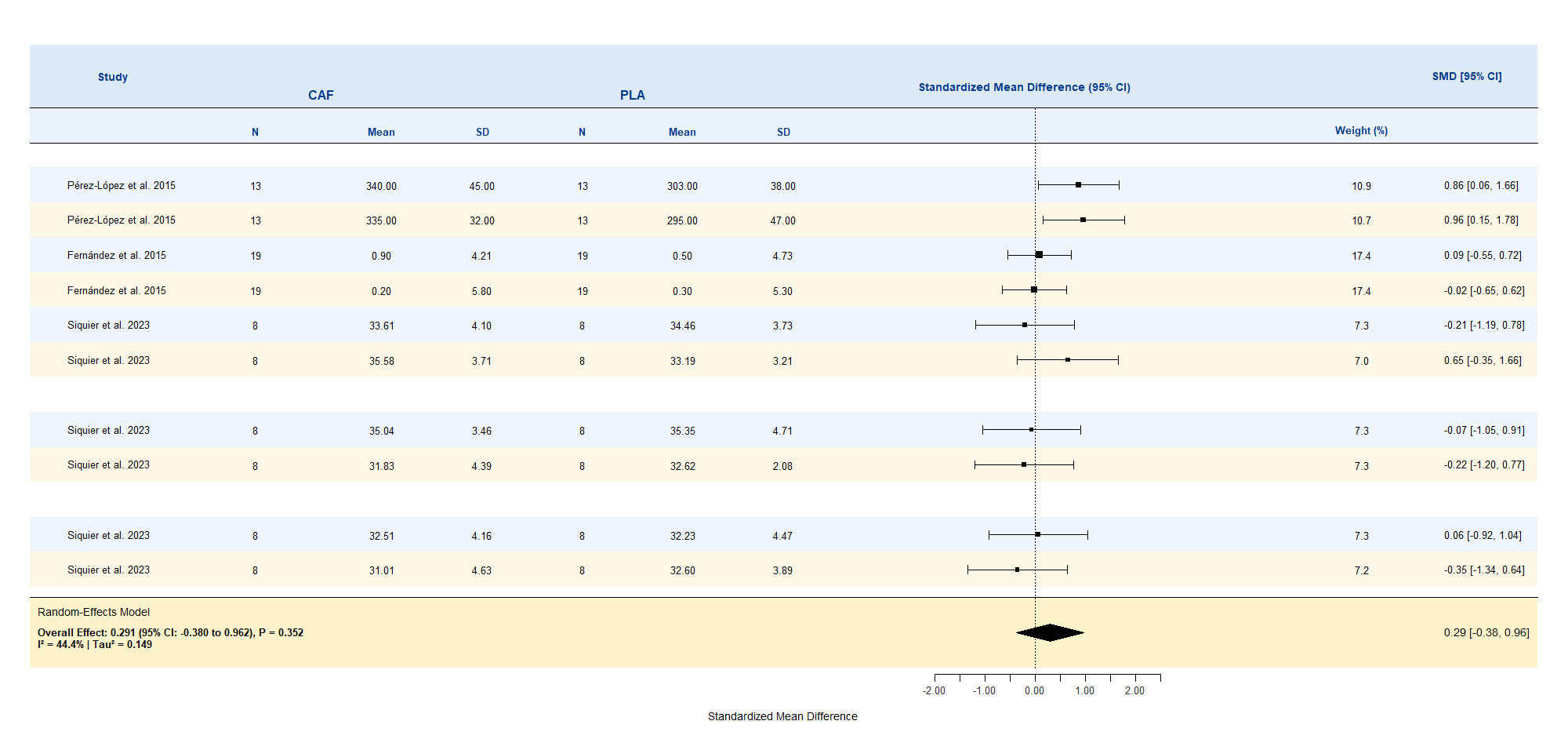
6.4 Handgrip strength performance**

**
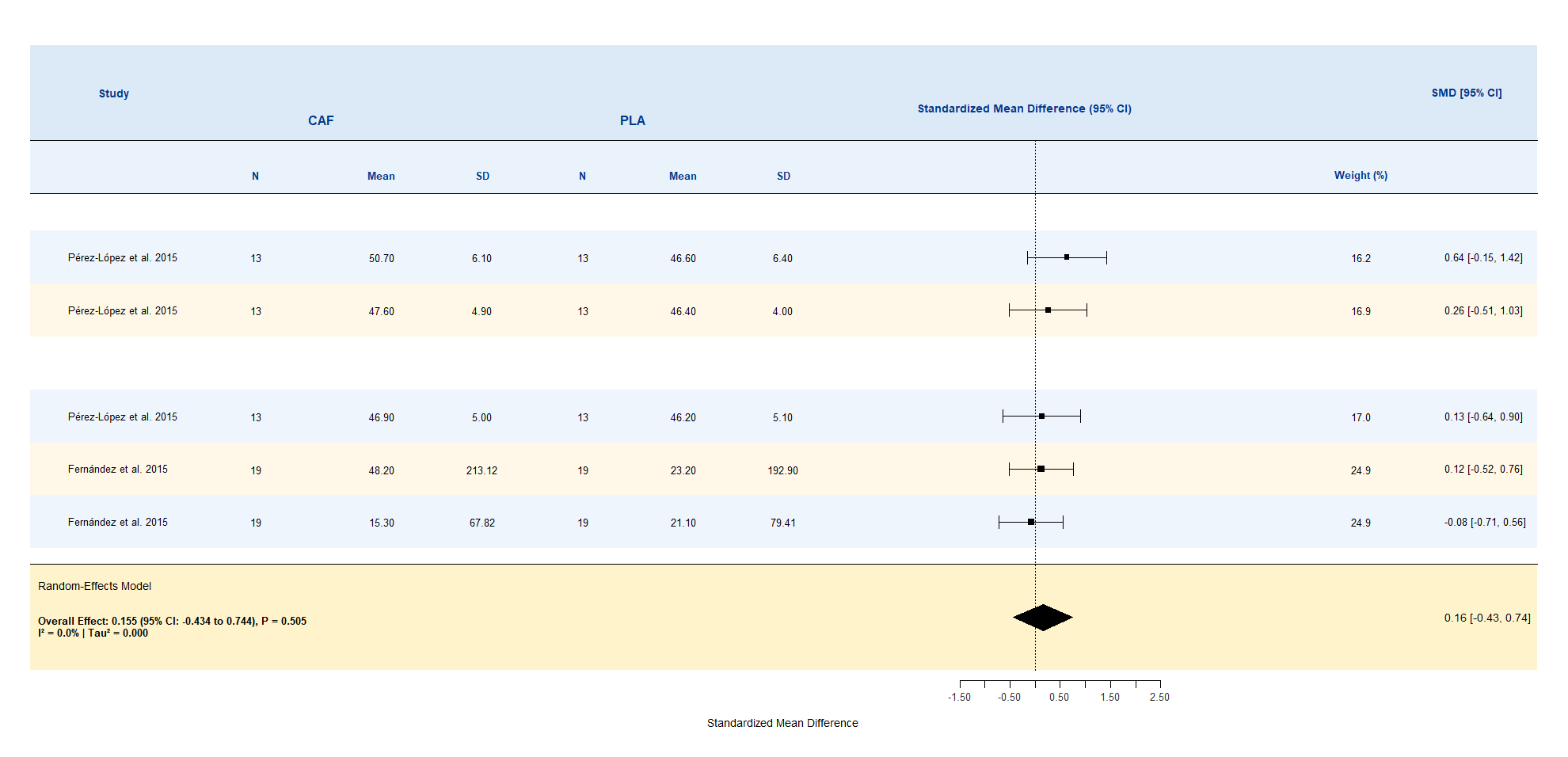
6.5 Power output**

**
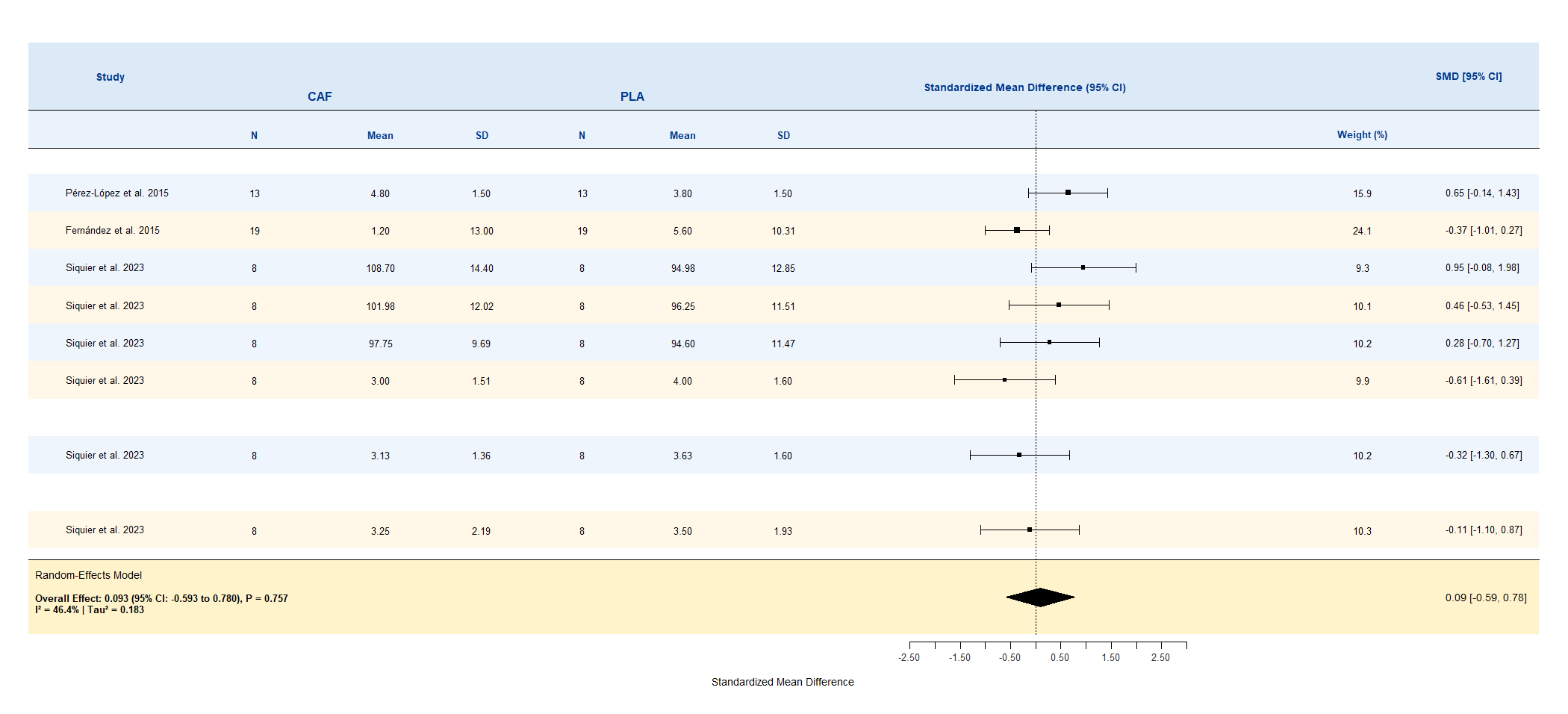
6.6 Fatigue-related outcomes**

**
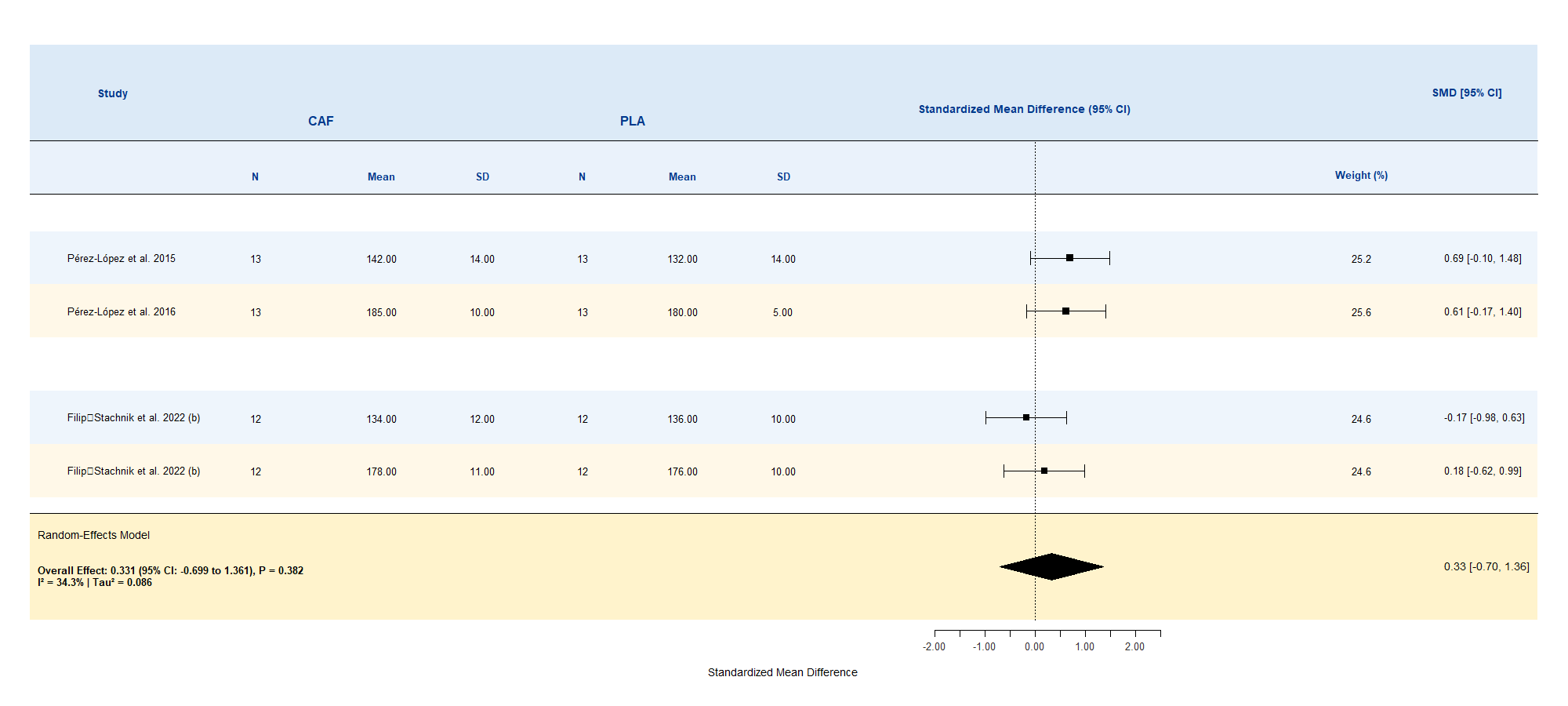
6.7 Heart Rate**
